# Sustained Type I interferon signaling as a mechanism of resistance to PD-1 blockade

**DOI:** 10.1038/s41422-019-0224-x

**Published:** 2019-09-03

**Authors:** Nicolas Jacquelot, Takahiro Yamazaki, Maria P. Roberti, Connie P. M. Duong, Miles C. Andrews, Loic Verlingue, Gladys Ferrere, Sonia Becharef, Marie Vétizou, Romain Daillère, Meriem Messaoudene, David P. Enot, Gautier Stoll, Stefano Ugel, Ilaria Marigo, Shin Foong Ngiow, Aurélien Marabelle, Armelle Prevost-Blondel, Pierre-Olivier Gaudreau, Vancheswaran Gopalakrishnan, Alexander M. Eggermont, Paule Opolon, Christophe Klein, Gabriele Madonna, Paolo A. Ascierto, Antje Sucker, Dirk Schadendorf, Mark J. Smyth, Jean-Charles Soria, Guido Kroemer, Vincenzo Bronte, Jennifer Wargo, Laurence Zitvogel

**Affiliations:** 1 INSERM U1015, Gustave Roussy, 114 rue Edouard Vaillant, 94805 Villejuif Cedex, France; 2 Université Paris-Saclay, Le Kremlin-Bicêtre, France; 3 Institut de Cancérologie Gustave Roussy Cancer Campus (GRCC), 114 rue Edouard Vaillant, Villejuif, France; 4 Faculté de Médecine–Université Paris-Sud, Le Kremlin-Bicêtre, France; 5 CIC Biotherapie IGR Curie, CIC1428, Gustave Roussy Cancer Campus, Villejuif, France; 6 Department of Surgical Oncology, MD Anderson Cancer Center, Houston, TX USA; 7 Olivia Newton-John Cancer Research Institute, Heidelberg, VIC Australia; 8 School of Cancer Medicine, La Trobe University, Heidelberg, VIC Australia; 9 Drug Development Department (DITEP), Gustave Roussy, Université Paris-Sud, Université Paris-Saclay, Villejuif, France; 10 Metabolomics and Cell Biology Platforms, Gustave Roussy Cancer Campus, Villejuif, France; 11 INSERM U1138, Centre de Recherche des Cordeliers, Paris, France; 12 Equipe 11 labellisée par la Ligue contre le Cancer, Centre de Recherche des Cordeliers, Paris, France; 13 Université Pierre et Marie Curie, Paris, France; 14 Université Paris Descartes, Sorbonne Paris Cité, Paris, France; 15 Department of Medicine, Verona University Hospital, Verona, Italy; 16 Istituto Oncologico Veneto IOV-IRCCS, Padova, Italy; 17 Immunology in Cancer and Infection Laboratory, QIMR Berghofer Medical Research Institute, Herston, QLD Australia; 18 School of Medicine, University of Queensland, Herston, QLD Australia; 19 INSERM, U1016, Institut Cochin, Paris, France; 20 CNRS, UMR8104 Paris, France; 21 Melanoma Cancer Immunotherapy and Innovative Therapy Unit, Istituto Nazionale Tumori IRCCS Fondazione “G. Pascale”, Napoli, Italy; 22 Department of Dermatology, University Hospital, University Duisburg-Essen, Essen, Germany & German Cancer Consortium (DKTZ), Heidelberg, Germany; 23 Pôle de Biologie, Hôpital Européen Georges Pompidou, AP-HP, Paris, France; 24 Department of Women’s and Children’s Health, Karolinska Institute, Karolinska University Hospital, Stockholm, Sweden; 25 Department of Genomic Medicine, The University of Texas MD Anderson Cancer Center, Houston, TX USA; 26 Present Address: Immunology Division, The Walter and Eliza Hall Institute of Medical Research, Melbourne, VIC Australia

**Keywords:** Tumour immunology, Cancer microenvironment

## Abstract

PD-1 blockade represents a major therapeutic avenue in anticancer immunotherapy. Delineating mechanisms of secondary resistance to this strategy is increasingly important. Here, we identified the deleterious role of signaling via the type I interferon (IFN) receptor in tumor and antigen presenting cells, that induced the expression of nitric oxide synthase 2 (NOS2), associated with intratumor accumulation of regulatory T cells (Treg) and myeloid cells and acquired resistance to anti-PD-1 monoclonal antibody (mAb). Sustained IFNβ transcription was observed in resistant tumors, in turn inducing PD-L1 and NOS2 expression in both tumor and dendritic cells (DC). Whereas PD-L1 was not involved in secondary resistance to anti-PD-1 mAb, pharmacological or genetic inhibition of NOS2 maintained long-term control of tumors by PD-1 blockade, through reduction of Treg and DC activation. Resistance to immunotherapies, including anti-PD-1 mAb in melanoma patients, was also correlated with the induction of a type I IFN signature. Hence, the role of type I IFN in response to PD-1 blockade should be revisited as sustained type I IFN signaling may contribute to resistance to therapy.

## Introduction

Major conceptual advances in cancer biology have been made over the past decade.^1–3^ The understanding that immune responses are routinely generated against tumor-specific neoantigens resulting from cancer–associated mutations^4–8^ and commonly suppressed by immunosuppressive tumor microenvironments (TME) has led to the development of effective immunotherapies aimed at provoking immune control against tumor progression.^9,10^ Cancer immunotherapy has resulted in remarkable success in the treatment of a variety of hematological and solid metastatic malignancies such as melanoma, lung, bladder, kidney, Hodgkin’s lymphoma, B cell acute lymphocytic leukemia, hepatocellular carcinoma, Merkel-cell carcinoma and head and neck tumors.^11–16^ To date, therapies that block inhibitory signaling molecules expressed by T lymphocytes (so called “immune checkpoints”) during the initial priming phase (in the draining lymph nodes) or the effector phases (in tumor beds) of adaptive anticancer immune responses have resulted in the greatest clinical benefit.^16,17^ A paradigm of this success has been the use of monoclonal antibodies (mAb) targeting Programmed cell death 1 (PD-1) (expressed by activated/exhausted T cells) or its ligand PD-L1 (commonly expressed by cancer cells or cells of the TME).^16,18^ By releasing these molecular brakes, such mAbs reinstate the anticancer adaptive arm of the immune response.

While PD-1 blockade represents the most effective first line therapy in *BRAF* wildtype melanoma and the best option in first-line non-small cell lung cancer, when combined with platinum-based chemotherapy, about 60–70% of tumors do not clinically benefit from this treatment and exhibit primary resistance to this therapeutic strategy.^19,20^ Primary resistance has been attributed to several factors including low tumor mutational burden and poor intrinsic antigenicity of tumor cells;^5,6^ defective antigen presentation and priming phase;^21^ limited tumor infiltration related to exhausted T cell functions;^2^ CSF1-dependent tumor associated macrophage accumulation;^22^ and immunosuppressive metabolic pathways related to adenosine and indoleamine-2,3-dioxygenase (IDO).^2^ Importantly, genomic defects in IFNγ signaling pathway genes have been found to provide a primary mechanism leading to resistance to therapy targeting cytotoxic T-lymphocyte-associated protein 4 (CTLA-4), including in melanoma.^23^

More recently, specific mechanisms of secondary resistance to chronic inhibition of PD-1 receptors have been described in about 25% of melanoma patients.^24–26^ A subset of melanoma patients who progressed despite an initial response to therapy with pembrolizumab, which targets PD-1, displayed either loss-of-function mutations in Janus kinases *JAK1* or *JAK2*, leading to reduced sensitivity to the anti-proliferative effects of IFNs, decreased phosphorylation of STAT1, or a truncating mutation in the gene β2 microglobulin, resulting in defective antigen presentation due to prevention of folding and transport of MHC class I molecules to the cell surface for T cell recognition of tumor cells.^26^ Similar alterations in β2 microglobulin have been found in about 30% of non-responders treated with a combination of anti-PD-1 and anti-CTLA-4.^27^ In addition to the natural selection of heritable genetic (or epigenetic) traits previously described,^28,29^ other acquired resistance mechanisms have also been reported such as the IFNγ-inducible expression of PD-L1;^30,31^ TNFα-induced loss of antigenic variants;^32^ and TCR-dependent upregulation of additional exhaustion markers on activated T lymphocytes including T-cell immunoglobulin and mucin-domain containing-3 (Tim3), lymphocyte activation gene 3 (Lag3), T cell immunoreceptor with Ig and ITIM domains (TIGIT), B and T cell lymphocyte attenuator (BTLA), and V-domain Ig suppressor of T cell activation (VISTA).^2,11,24^

Prompted by the relatively short duration of tumor control following iterative administrations of anti-PD-1 mAb in tumor bearing mice, we identified a detrimental role of interferon-α/β receptor (IFNAR) signaling in both CD45^−^ and CD45^+^ intratumoral subsets inducing the expression of nitric oxide synthase 2 (NOS2). This was associated with the reduction of intratumor polyfunctional cytotoxic T lymphocytes (CTLs), increased regulatory T cells and myeloid cells leading to acquired resistance to anti-PD-1 mAb-based therapy. A chronic PD-1 blockade progressively induced IFNγ and IFNβ transcription in the TME, with both IFNs in turn triggering PD-L1 and NOS2 expression on both tumor cells and leukocytes. Anti-PD-1 mAb-sensitive tumors exhibited lower endogenous levels of NOS2, and progressively lost TME-derived IFNβ production. In contrast, resistant tumors, presenting high NOS2 expression at baseline, sustained their IFNβ release, the main trigger of NOS2 upregulation and maintenance. While PD-L1 was not involved in secondary resistance to anti-PD-1 mAb, reducing NOS2 with L-NAME or a genetic knockout improved long-term tumor control by PD-1 blockade in several tumor models. Finally, we highlight the potential clinical significance of this pathway in secondary resistance to first-line therapies (including PD-1 blockade) in advanced melanoma patients.

## Results

### Resistance of murine tumors to PD-1 blockade

**Fig. 1:**
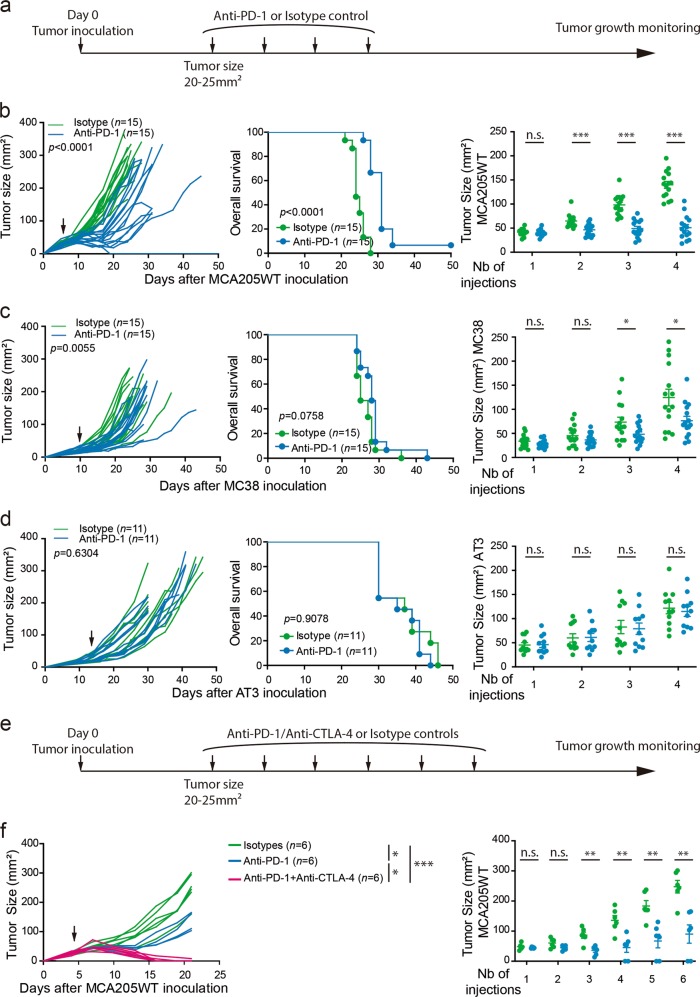
“Sensitive”, “eventually resistant” and “innately resistant” tumor models to PD-1 blockade. **a** and **e** Therapeutic antitumor protocols based on anti-PD-1 or its isotype control. When tumors reached 20–25 mm^2^ (indicated by an arrow), anti-PD-1 mAb (or its isotype control) alone **a** or together with anti-CTLA-4 mAb **e** were administered i.p. every 3 days for 4 **a** to 6 **e** injections (as described in Material and Methods). **b**–**d**, **f** MCA205WT sarcoma **b**, **f**, MC38 colon **c** or AT3 breast **d** cancer cells were injected subcutaneously. Tumor growth kinetics (left panels), survival curves (middle panels) and tumor sizes after sequential injections of isotype or anti-PD-1 mAb (right panels) are depicted. Each line or dot corresponds to one animal. Each graph represents 1 experiment **f**, a pool of 2 **d** to 3 **b**, **c** experiments with 5–6 animals per group and per experiment. For tumor growth and Kaplan–Meier curves, statistical analyses were performed using the specific software detailed in the Material and Methods. Unpaired t-tests were used in **b**, **c**, **d** and **f**, right panels. *p < 0.05, **p < 0.01, ***p < 0.001, *n*.s.: not significant. Means ± SEM are represented

As resistance to anti-PD-1 treatment is a major clinical issue, a better understanding of the cellular and molecular mechanisms involved is critical. While, many mechanisms have been described to be responsible for primary resistance,^2,5,6,21,22^ the mechanisms underlying secondary or acquired resistances to anti-PD-1 therapies are still not well understood. Secondary or acquired resistance to anti-PD-1 blockade is defined as a loss of treatment efficacy while being on therapy, resulting in tumor growth and progression. To tackle this question, we treated four established transplantable tumor models syngeneic to C57BL/6 mice with 4 biweekly intraperitoneal (i.p.) administrations of anti-PD-1 mAb (clone RMP1–14) (Fig. 1a). While the MCA205WT sarcoma remained sensitive to 4 doses of PD-1 blockade over 12 days (Fig. 1b; Supplementary information, Fig. S1b), hosts bearing MCA205OVA and MC38 colon cancers were eventually resistant as initial treatments slightly reduced their tumor growth kinetics (Fig. 1c; Supplementary information, Fig. S1a, c and e). However, AT3 breast tumors were primarily resistant to mAb, exhibiting no benefit in tumor control or overall survival (Fig. 1d; Supplementary information, Fig. S1d).^33^ Whilst tumor growth in the sensitive (MCA205WT) model displayed a rebound after the period of active anti-PD-1 therapy, we showed that prolonging the administration of anti-PD-1 mAb from 4 to 6 doses failed to extend the efficacy of therapy, culminating in overt tumor progression in the majority of animals (Fig. 1e, f; Supplementary information, Fig. S1f). However, concurrent CTLA-4 blockade in this model could prevent secondary resistance to this treatment (Fig. 1e, f).

We then performed a comprehensive immunological phenotyping of splenocytes and intratumor cells (Supplementary information, Fig. S2a, b) after 4 administrations of anti-PD-1 mAb in sensitive (MCA205WT sarcoma), eventually resistant (MCA205OVA sarcoma) and innately resistant (AT3 breast cancer) tumor models. Surrogate hallmarks of anticancer efficacy were observed consistently in all treated mice bearing MCA205WT but only selectively in responding mice harboring MCA205OVA cells reflected by intratumor leukocyte influx, the upregulation of ICOS expression on CD8^+^ TILs—relative to anti-PD-1 non-responders (NR) in MCA205OVA mice—the increase in polyfunctional (IFNγ^+^TNFα^+^) CTLs and an increased CD8^+^/CD4^+^FOXP3^+^ ratio (in MCA205 only) (Supplementary information, Fig. S2c, d). Splenic immune parameters failed to correlate with the sustained success of PD-1 blockade (Supplementary information, Fig. S2a).

Hence, we took advantage of the contrast between relatively sensitive MCA205WT (although showing a late escape from the PD-1 blockade) and MC38 tumors that promptly overcome PD-1 blockade to scrutinize the underlying mechanisms of delayed resistance to PD-1 blockade.

### Interrogation of host and tumor IFNAR signaling pathways in resistance to PD-1 blockade

**Fig. 2:**
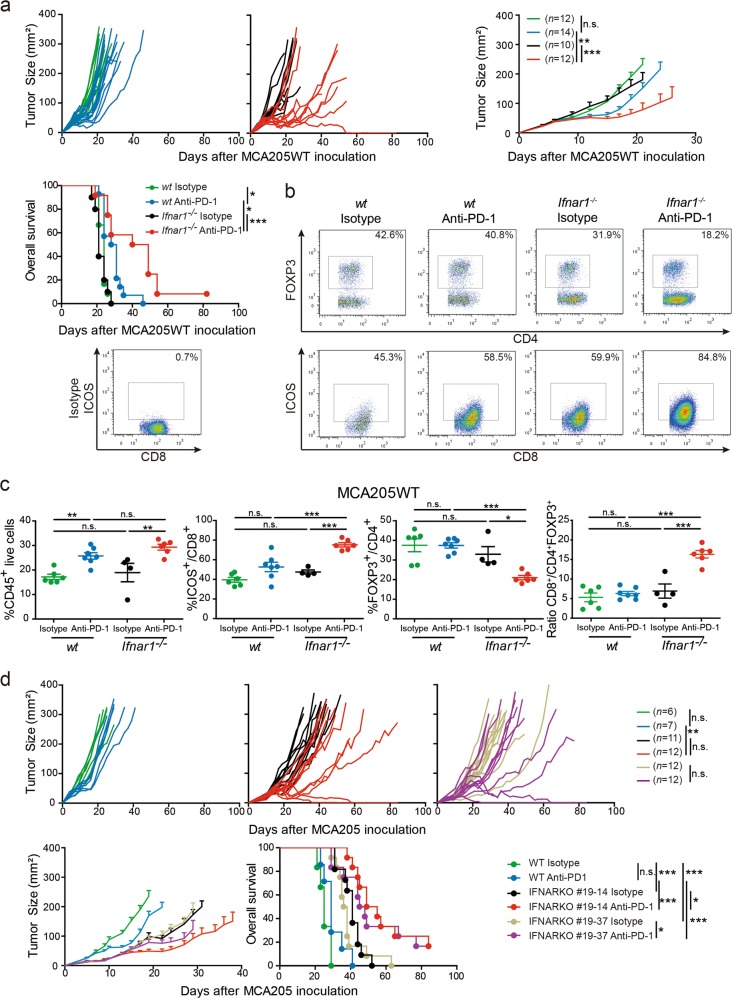
Host and tumor IFNAR1 involved in secondary resistance to PD-1 blockade. **a** MCA205WT growth kinetics (top panels) and Kaplan–Meier survival curves (bottom panel) of WT and *Ifnar1*^−/−^ C57BL/6 mice treated with anti-PD-1 mAb (or its isotype control) as described in Fig. 1a. **b** and **c** Representative gating strategy **b** and flow cytometry analyses **c** of tumor-infiltrating leukocytes after 4 injections and the proportions of CD45^+^ (left panel), ICOS^+^ cells in the CD8^+^T cell gate and FOXP3^+^ cells in the CD4^+^T cell gate (middle panels) and the ratio CD8^+^/CD4^+^FOXP3^+^ (right panel) are depicted. **d** Tumor growth kinetics and Kaplan–Meier survival curves of WT mice inoculated with two different clones of *Ifnar1*^−/−^ MCA205. Each line or dot represents one animal. The graphs represent 1 experiment (**c**) or depict pooled data from 2 **a** and **d** independent experiments encompassing 4–7 mice/group. For tumor growth and Kaplan–Meier curves, statistical analyses were performed using the specific software detailed in the Material and Methods. ANOVA statistical tests and pairwise comparisons with Bonferroni adjustment were used in **c**. *p < 0.05, **p < 0.01, ***p < 0.001, n.s.: not significant. Means ± SEM are represented

A large body of literature strongly supports the key role of the IFNAR signaling pathway in controlling anticancer immunosurveillance, and dictating the success of chemotherapy and radiotherapy-induced immune responses, even against MHC class I-deficient tumors that are innately resistant to PD-1 blockade.^34–37^ The expression of IFNAR1 was comparable between the MCA205, MC38 and AT3 tumor cell lines (Supplementary information, Fig. S3). They were all sensitive to in vitro stimulation with IFNα or IFNγ, resulting in MHC class I and/or PD-L1 upregulation, as well as CXCL10 secretion in all cell lines at least initially sensitive to PD-1 inhibition (Supplementary information, Fig. S3). We next investigated the role of IFNAR1 expressed by the host or intrinsically by tumor cells in affecting the sensitivity of MCA205WT tumors to anti-PD-1 mAb in vivo. While we saw no differences in tumor control based on IFNAR1 status in the absence of treatment, administration of anti-PD-1 mAb into *Ifnar1*^−/−^ mice extended tumor control and prolonged survival compared with that observed in wild type (WT) mice (Fig. 2a). Accordingly, the surrogate hallmarks of efficacy of PD-1 blockade were all markedly enhanced in the absence of a functional IFNAR1 host signaling pathway (Fig. 2b). Whilst the intratumor fraction of CD45^+^ cells was not significantly increased, augmented ICOS-expressing CTLs, together with reduced Treg infiltration and an increased CD8^+^/CD4^+^FOXP3^+^ ratio, were observed in *Ifnar1*^−/−^ compared with *Ifnar1*^+/+^ anti-PD-1-treated mice (Fig. 2c). Moreover, the therapeutic outcome of a PD-1 blockade was influenced by tumor cell expression of IFNAR1. In fact, two MCA205 clones,19–14 and 19–37,^38^ produced by nucleo-transfection with specific zinc finger nucleases (ZFN) causing targeted deletion of the *Ifnar1* gene were implanted into *Ifnar1* wild-type mice. These exhibited inherently reduced tumor growth kinetics and, more importantly, heightened response to anti-PD-1 mAb resulting in complete tumor eradication in up to 17% of mice (Fig. 2d).

**Fig. 3:**
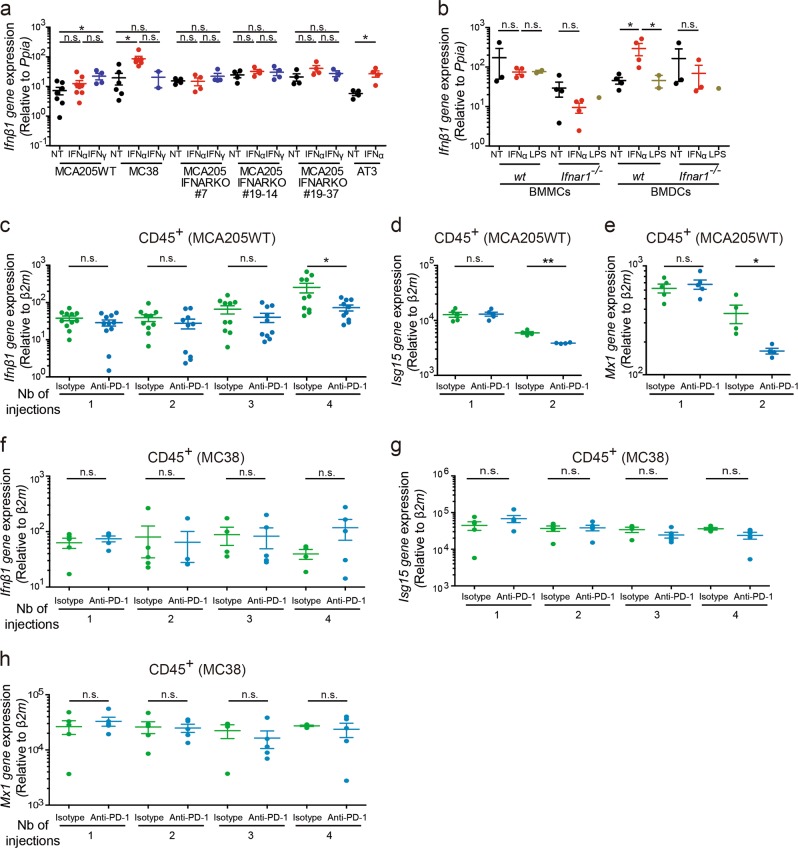
Sources and kinetics of Type I IFN in the TME during PD-1 inhibition. **a** and **b** In vitro assays. Relative expression of *Ifnβ1* quantified by qRT-PCR following stimulations of various tumor cell lines or BMDCs and BMMCs with IFNα, IFNγ or LPS. Each dot represents one sample and graphs represent 1 experiment or are the pool of 2 to 3 independent experiments including biological replicates for each experiment. Unpaired t-tests were used to compare 2 groups. ANOVA statistical tests and pairwise comparisons with Bonferroni adjustment were adopted for more than 2 groups. **c**–**h** In vivo studies. Flow cytometry sorting of CD45^+^ live fractions from the TME of MCA205WT **c**–**e** or MC38 **f**–**h** tumors 48 h after 1, 2, 3 or 4 i.p. administrations of anti-PD-1 (or isotype control) mAb. Relative expression of *Ifnβ1* **c** and **f** and IFN-sensitive gene products **d**, **e**, **g**, **h** quantified by qRT-PCR. Unpaired t-tests were used to compared transcription levels between the anti-PD-1 and isotype control treated groups for each time point. Each dot represents 1 mouse with 5 mice per time point per experiment. Graphs represent 1 representative experiment out of 2–3 independent experiments (MC38, time points 1 and 2, **d**, **e**), 1 experiment (MC38, time points 3 and 4) or are the pool of 2–3 independent experiments **c**. *p < 0.05, **p < 0.01, n.s.: not significant. Mean ± SEM are represented

To understand the autocrine regulation of type I IFN release by either tumor or host cells, we subjected tumor cell lines and bone marrow-derived myeloid cells (BMMCs) or bone-marrow derived dendritic cells (BMDCs) to a 24-h stimulation with recombinant IFNα. The relatively anti-PD-1-resistant MC38 and AT3 cells significantly upregulated *Ifnβ1* transcription in response to IFNα whilst no significant change was observed in the anti-PD-1-sensitive MCA205WT or *Ifnar1*^−/−^ sarcoma clones (Fig. 3a). Consistent with other reports,^34–36^ BMDCs exhibited positive feedback of type I IFN expression in an IFNAR1-dependent manner, in contrast to BMMCs (Fig. 3b). Along the course of PD-1 blockade, we observed that both intratumor CD45^+^ and CD45^−^ cell subsets sorted from sensitive tumors (MCA205WT) tended to lose the expression of some interferon-stimulated gene (ISG) gene products in vivo (Fig. 3c–e; Supplementary information, Fig. S4a–c). This was in contrast to resistant tumors (MC38) that tended to maintain *Ifnβ1*, *Mx1 and Isg15* gene expression (Fig. 3f–h; Supplementary information, Fig. S4d–f).

Taken together, the IFNAR1 signaling pathways comes into play in tumor cells and leukocytes to subvert tumor growth control by anti-PD-1 mAb, especially when type I IFNs are produced by both CD45^+^ and CD45^−^ cellular compartments.

### PD-1 blockade broadly induced PD-L1 upregulation with no influence on tumor progression

To further explore the effect of PD-L1 expression in cancer resistance to IFNγ producing CTLs that express PD-1,^31^ we investigated the regulation of PD-L1 transcription in all tumor cell lines by qRT-PCR in vitro. All cell lines tended to upregulate *Pdl1* gene transcription similarly after IFNγ stimulation, while type I IFN stimulation was IFNAR1-dependent (Supplementary information, Fig. S5a). We further analyzed the expression of PD-L1 on the cell surface of tumor and immune cells in sensitive MCA205WT or resistant AT3 tumors after 4 injections using flow cytometry (Supplementary information, Fig. S5b–e). As expected, both tumor infiltrating myeloid cells and the CD45^−^ fraction acquired PD-L1 expression. Cell subsets from sensitive tumors expressed much higher PD-L1 levels than those from resistant tumors (Supplementary information, Fig. S5b–d). The expression of PD-L1 on CD45^−^ cells was more pronounced in *Ifnar1*-deficient hosts and this was associated with improved anti-PD-1 therapeutic efficacy (Fig. 2a; Supplementary information, S5e).

**Fig. 4:**
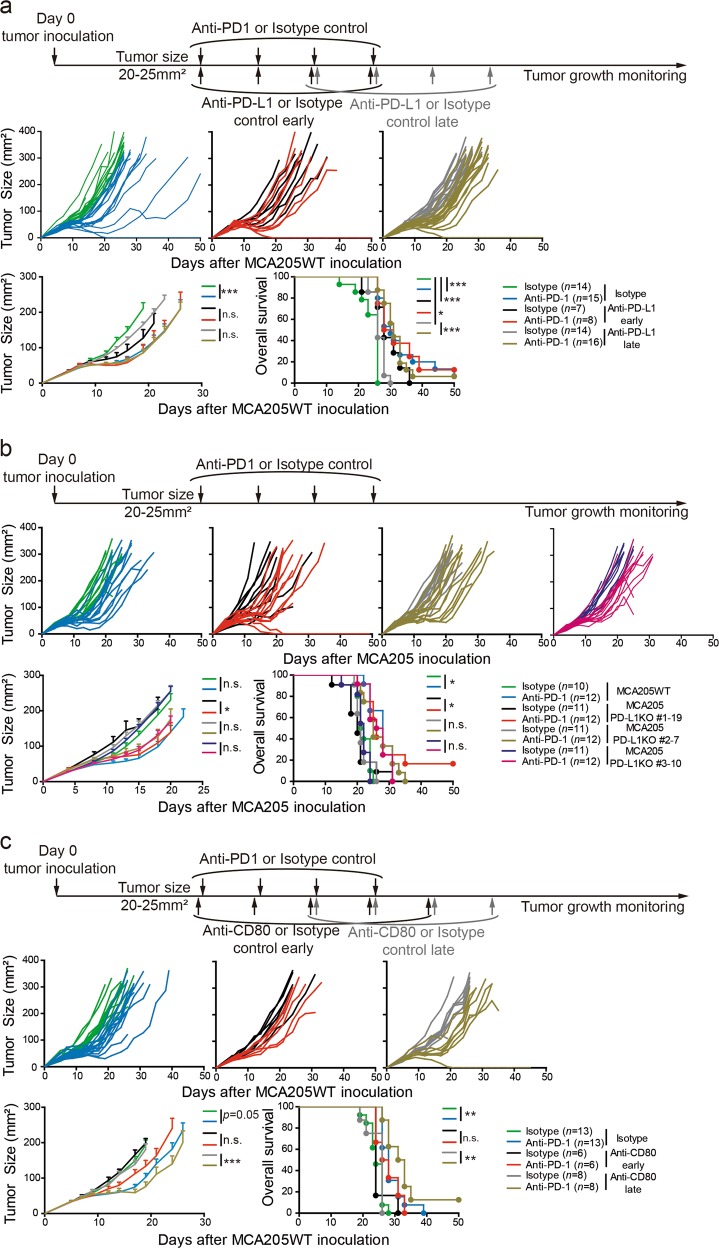
Secondary resistance to PD-1 blockade is PD-L1 independent. **a** Effects of neutralizing anti-PD-L1 mAb co-administered early or late following initiation of anti-PD-1 mAb in MCA205WT tumor bearers. **b** Effects of anti-PD-1 mAb treatment on tumor growth against MCA205WT versus two different clones of *Pd-l1*^−/−^ MCA205. **c** Equivalent schema as in **a** using anti-CD80 mAb. Each line or dot represents 1 animal. Mean ± SEM are represented. Tumor growth kinetics and Kaplan–Meier survival curves are shown. The graphs depict tumor growth kinetics of 1 (**a**, anti-PD-L1 early; **c**, anti-CD80 early and late) or 2 independent experiments encompassing 5–8 mice/group and per experiment. Statistical analyses were performed using the specific software detailed in the Material and Methods. *p < 0.05, **p < 0.01, ***p < 0.001, n.s.: not significant

Despite these observations, four lines of experimental evidence underscored the lack of functional relevance of PD-L1 upregulation in secondary resistance to anti-PD-1 mAb. First, concomitant or sequential co-blockade of PD-1 and PD-L1 receptor/ligand failed to improve tumor control (Fig. 4a). Second, the anti-PD-1 mAb did not exhibit more sustained antitumor effects against three independent clones of PD-L1-deficient sarcoma (Fig. 4b). Third, co-inhibition of PD-1 and CD80, the alternate receptor for PD-L1, using anti-CD80 mAb or a CTLA-4-Ig fusion protein, Abatacept, failed to control tumor escape or prolong survival over PD-1 blockade alone (Fig. 4c; Supplementary information, Fig. S6). Finally, the significant inverse correlation observed between tumor size and endogenous levels of PD-L1 expression by CD45^−^ cells as measured by MFI, in the absence of therapy (Supplementary information, Fig. S6b), was not seen following treatment with anti-PD-1 (Supplementary information, Fig. S6c). This suggested that PD-L1 expression was more likely to be an indirect hallmark of natural anticancer immunosurveillance, rather than a surrogate marker of secondary resistance of PD-1 blockade. Additionally, this anti-correlation was also not observed in *Ifnar1*-deficient tumor clones (not shown), corroborating the intrinsic role of type I IFN in natural immunosurveillance.^39^

These data suggest that type I IFN contributes to the upregulation of PD-L1 expression in the TME during the course of anti-PD-1 therapy, likely reflecting the extent of underlying adaptive anticancer immune responses. However, PD-L1 upregulation per se is unlikely to be the cause of secondary resistance to PD-1 blockade in our pre-clinical model.

### IFNs-induced NOS2 compromises sustained responses to PD-1 blockade

**Fig. 5:**
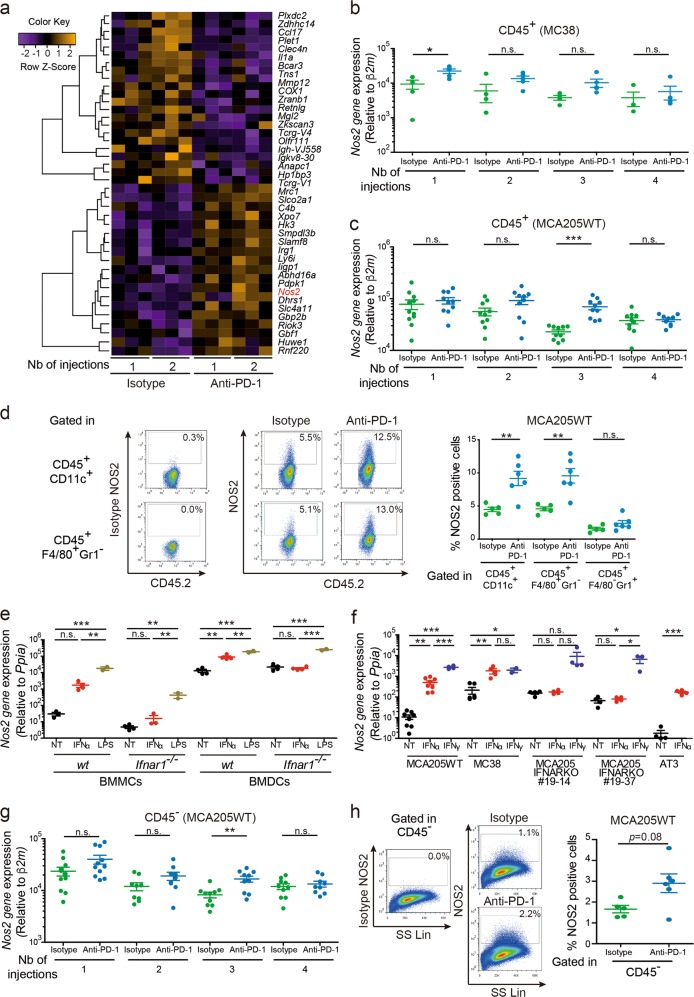
Type I IFN-induced Nos2 expression post- PD-1 blockade in the TME. **a** Microarray analysis and qRT-PCR analyses of TILs in MC38 during PD-1 blockade. CD45^+^ cells from MC38 tumors were cell-sorted 48 h post-1, 2 injections of anti-PD-1 or isotype control mAbs to perform trancriptomic analyses. Heat-map depicting the shared significant downregulated and upregulated genes in the anti-PD-1 treated groups compared with the isotype control treated groups across time points 1 and 2. **b** Relative expression of *Nos2* in CD45^+^ cells from MC38 tumors 48 h after 1, 2, 3 and 4 injections of anti-PD-1 or Isotype mAbs using qRT-PCR analyses. **c** and **g** Same as **b** with MCA205WT tumor bearers after treatment with 1, 2, 3 and 4 injections of mAbs evaluated in both CD45^+^ **c** and CD45^−^ **g** fractions. **d** and **h** Representative gating strategy and flow cytometric analyses of NOS2 protein expression in CD45^+^ cells **d** and in the CD45^-^ fraction **h**, 48 h after the fourth injection of anti-PD-1 or its isotype control mAbs. **e**, **f** Relative expression of *Nos2* quantified by qRT-PCR following stimulations of BMDCs and BMMCs **e** or various tumor cell lines **f** with either IFNα, IFNγ or LPS. Each dot corresponds to one stimulated sample or 1 mouse with 2 or more biological replicates per experiment and 5 mice per group per time point per experiment. Graphs depict 1 experiment (**b**, time points 3 and 4, **d** and **h**), are representative of 1 experiment out of 2–3 performed (**b**, time points 1 and 2), or are the pool of 2–3 independent experiments **c**, **e**–**g** including biological replicates for each experiment. Unpaired *t*-tests were used to compare two groups (**b**–**d**, **f** for AT3 tumor model and **g**, **h**). ANOVA statistical tests and pairwise comparisons with Bonferroni adjustment were adopted for more than 2 groups **e**, **f**. *p < 0.05, **p < 0.01, ***p < 0.001, n.s.: not significant. Means ± SEM are represented

The type I and type II IFN receptor signaling pathways culminate in ISRE and GAS-inducible promoter transcriptional activity of various ISG including the well-described inducible nitric oxide synthase (*Nos2*) gene product.^40–42^ We contrasted a microarray analysis of all protein-coding transcripts expressed in CD45^+^ fractions of established MC38 tumors after 1 and 2 administrations of isotype control versus blocking anti-PD-1 mAb (Fig. 5a, Supplementary information, Table S1). *Nos2* was the third most upregulated gene product after PD-1 blockade in TILs (Table S1). We confirmed these findings by qRT-PCR on the CD45^+^ fraction, which showed a prompt upregulation of *Nos2* expression in PD-1 treated tumors 48 h after the 1st injection (Fig. 5b), preceding the clinical observation of anti-PD-1 resistance (Fig. 1c; Supplementary information, S2c). This upregulation was not observed in the CD45^−^ fraction (not shown). In MCA205WT, *Nos2* was significantly upregulated after 3 anti-PD-1 injections (Fig. 5c) and this, once again, preceded the therapy resistance observed after 4 injections (Fig. 1b, 1f; Supplementary information, Fig. S1f). Surprisingly, *Nos2* expression tended to decrease after 4 injections (Fig. 5c). However, at the protein level, flow cytometry analyses performed after 4 injections of mAb revealed two-fold higher NOS2 expression in CD11c^+^ as well as F4/80^+^/Gr1^−^ fractions of CD45^+^ tumor infiltrating cells from anti-PD-1-treated mice compared with isotype-treated controls (Fig. 5d). qRT-PCR and flow cytometric analyses showed that type I IFN (or LPS)-stimulated BMDCs expressed higher levels of *Nos2* than BMMCs (Fig. 5e; Supplementary information, Fig. S7). Tumor cells upregulated *Nos2* expression in response to type I or II IFN stimulation in vitro (Fig. 5f). In addition, the CD45^−^ fraction from MCA205WT tumors in vivo also expressed higher levels of *Nos2* at later stages of PD-1 blockade (Fig. 5g) with a trend towards higher NOS2 protein levels in mAb-treated tumor cells compared with isotype (Fig. 5h). In contrast, the neuronal *Nos1* and the endothelial *Nos3* gene transcripts were not significantly regulated by type I IFNs, in either tumor cells or leukocytes (Supplementary information, Fig. S8). We also investigated the expression of *Arginase 1* (*Arg 1*), which is known to be involved in immunosuppressive functions as a second pathway regulating the arginine availability in the TME.^43,44^ Contrary to *Nos2*, type 2 cytokines IL-4, IL-10, IL-13 or TGFβ stimulation, but not IFNγ, induced *Arg 1* expression in murine macrophages.^44,45^ In line with previous findings,^45^ *Arg 1* expression was not directly modulated by IFNs in BMDCs or BMMCs, or in tumor cells (Supplementary information, Fig. S7, Fig. S9a, b). Moreover, its in vivo expression was not modified by anti-PD-1 therapy in either CD45^+^ or CD45^−^ fractions (Supplementary information, Fig. S9c, d). Finally, these results were corroborated using mice deficient in *Arginase 1* expression in Tie2 positive cells demonstrating that the anti-tumor efficacy of the PD-1 blockade was not improved when arginase 1 was absent. (Supplementary information, Fig S9d).

Taken together, these findings indicate that *Nos2* expression precedes the resistance to anti-PD-1 blockade. Furthermore, PD-1 therapy further increases NOS2 expression which is regulated by type I (and type II) IFNs in both tumor cells and the tumor infiltrating myeloid fractions.

### Critical role of the IFNAR/NOS2 signaling pathway in secondary resistance to PD-1 blockade

**Fig. 6:**
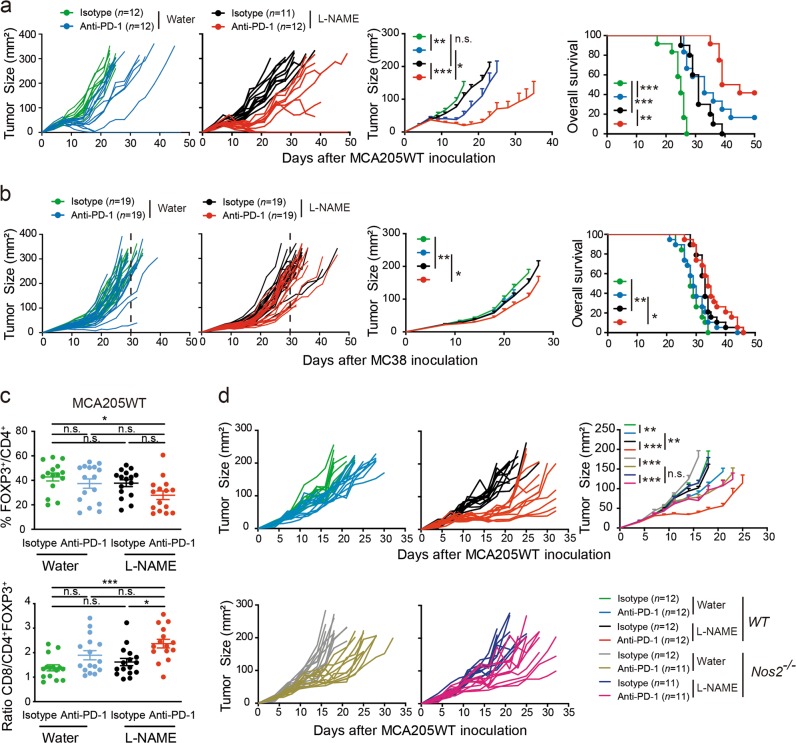
Pharmacological inhibition of NOS2 ameliorated the efficacy of PD-1 blockade in various tumor models. Concomitant blockade and inhibition of PD-1 and NOS2 with L-NAME in established MCA205WT **a** and MC38 **b** tumors. Tumor growth kinetics (left and middle panels) and survival curves (right panels) are depicted for each group. L-NAME treatment (1 g/L) was started one day prior to anti-PD-1 infusion and was maintained until the end of the experiment. The graphs depict tumor growth kinetics of a pool of 2–3 independent experiments encompassing 5–9 mice per group and per experiment. **c** Flow cytometry analyses of TILs after 4 injections focusing on the proportion of FOXP3^+^ cells among CD4^+^ T cells and the ratio of CD8^+^ T cells/CD4^+^ FOXP3^+^ Treg cells are depicted. Each dot represents 1 animal and graphs depict the pool of 3 independent experiments. Means ± SEM are represented. Statistical analyses were performed using ANOVA statistical tests and pairwise comparisons with Bonferroni adjustment. **d** Effect of L-NAME in anti-PD-1-treated MCA205WT tumors inoculated into WT or *Nos2*^*−/−*^ mice. The graphs depict a pool of 2 independent experiments including 5–7 mice/group and per experiment. Statistical analyses were performed using the specific software detailed in the Material and Methods. *p < 0.05, **p < 0.01, ***p < 0.001, n.s.: not significant

We next assessed the biological relevance of NOS2 function in adaptive resistance to PD-1 blockade. For this purpose, we treated MC38 colon cancers and MCA205WT sarcoma exhibiting early versus late resistance to anti-PD-1 mAb with the non-selective nitric oxide synthase inhibitor, Nω-Nitro-L-arginine methyl ester hydrochloride (L-NAME). In sensitive MCA205WT sarcoma, L-NAME induced a sustained efficacy of anti-PD-1 mAb, beyond day 40, associated with long-term survival (Fig. 6a). To a lesser extent, L-NAME also improved anti-PD-1 efficacy in MC38-bearing mice (Fig. 6b). Flow cytometric analyses of TILs revealed that L-NAME combined with an anti-PD-1 blockade tended to decrease tumor-infiltrating CD4^+^FOXP3^+^ regulatory T cells, and significantly increased the CD8^+^/CD4^+^FOXP3^+^ ratio in MCA205WT tumors (Fig. 6c).

Additionally, we took advantage of *Nos2*-deficient mice which we inoculated with MCA205WT tumor cells. This experimental set up revealed the critical role of NOS2-expressing host cells in the resistance to the anti-PD-1 blockade. In fact, we did not observe any amelioration of the antitumor efficacy of PD-1 blockade in combination with L-NAME in *Nos2*-deficient mice whereas this combination markedly increased the anti-PD-1 treatment efficiency in WT mice (Fig. 6d).

Altogether, IFNAR signaling in leukocytes led to deleterious *Nos2* expression contributing to progressive resistance to PD-1 blockade.

### Clinical relevance of the type I IFN/NOS2 pathway in patients treated with anti-PD-1 mAb

We then analyzed the regulation of *NOS2* gene expression by type I IFN in peripheral blood mononuclear cells (PBMC) from eight healthy volunteers (HV) and four melanoma (MEL) patients (Supplementary information, Fig. S10a, upper panel) together with TILs from seven stage III/IV MEL patients (Supplementary information, Fig. S10a, lower panel). As observed in mouse cells, we detected a significant upregulation of *NOS2* (but not *NOS3)* and *PDL1* gene expression in PBMCs but not in TILs at 24–60 h post-stimulation with type I IFN (Supplementary information, Fig. S10a).

We next examined NOS2 expression in tumor tissue in seven MEL patients treated with PD-1 blockade (patient characteristics detailed in Table S2). We carried out immuno-staining with an anti-NOS2 antibody upon paired biopsies, the first biopsy collected before treatment and the second biopsy collected during therapy or at relapse (Supplementary information, Fig. S10b). We observed a notable increase in NOS2 expression during late resistance to anti-PD-1 mAb in three out of seven cases (Supplementary information, Fig. S10c), all of which were also remarkable for having undetectable NOS2 immunostaining prior to therapy.

**Fig. 7:**
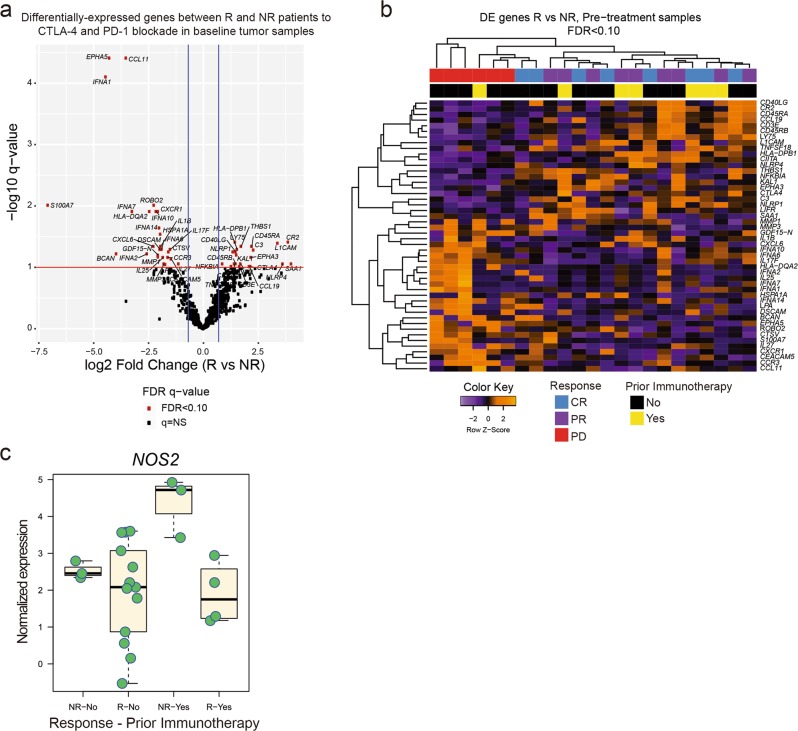
Type I IFN and NOS2 are associated with the secondary resistance of anti-PD-1 + anti-CTLA-4 therapy in patients. **a** Volcano plot of NanoString gene expression analysis in tumor biopsies harvested prior to initiation of combination anti-CTLA-4 and anti-PD-1 immune checkpoint blockade comparing responders (R) versus non-responders (NR). **b** Heatmap of differentially-expressed genes (FDR < 0.10) in pre-treatment samples comparing R versus NR patients, indicating RECIST-based best overall response (CR = complete response, PR = partial response, PD = progressive disease), and receipt of prior melanoma-directed systemic immunotherapy (cytokine, checkpoint blockade agent). **c** Boxplots of *NOS2* gene expression stratified by response to combination immune checkpoint blockade (R = responder, NR = non-responder) and prior immunotherapy status (Yes/No) demonstrating numerically higher *NOS2* levels in NR patients with prior immunotherapy exposure

To extend these initial findings, we investigated the gene transcripts associated with the response and resistance to PD-1 blockade immunotherapy with combined CTLA-4 co-blockade. We used a custom Nanostring panel to profile the expression of cancer/immune genes (Supplementary information, Table S3) in available pre-treatment tumors from 23 patients receiving combination anti-CTLA-4/PD-1 blockade for advanced melanoma (Andrews et al, manuscript in preparation) in a regimen that is often prescribed in the context of resistance to anti-PD-1 mAb. Differentially-expressed transcripts between responders (R; *n* = 17) and non-responders (NR; *n* = 6) revealed higher expression of several interferon-related genes (*IFNA1*, *IFNA2*, *IFNA6*, *IFNA7*, *IFNA10*, *IFNA14*) in NR tumors, consistent with our preclinical findings implicating type I IFN signaling in poor responses to immune checkpoint blockade (Fig. 7a). Also enriched in NR tumors were the inflammatory cytokine *IL1B*, and chemokine *CXCL6* which has been implicated in melanoma growth and metastasis.^46^ There was no obvious correlation between receipt of prior immunotherapy and response or expression level of type I IFN or *IFNAR1/2*-related genes, despite the majority of immunotherapy pre-treated patients (*n* = 5, 71%) having received checkpoint blockade agents for the treatment of metastatic disease (Fig. 7b). However, non-responding patients with a history of prior immunotherapy had numerically higher baseline levels of *NOS2*, but not *IFNAR1/2*, compared with immunotherapy pre-treated responders (Fig. 7c; Supplementary information, Fig. S11) suggesting a potential contribution of pre-activated *NOS2* to treatment failure.

Therefore, the type I IFN pathway is activated in the TME at diagnosis or during treatment with immunomodulators eventually leading to *Nos2* expression. This paves the way to resistance to cancer immunosurveillance.

## Discussion

By studying various tumor models exhibiting phenotypic traits of reduced, early or late resistance to a therapeutic antibody inhibiting the PD-1 immune checkpoint, we have unraveled IFNAR-induced NOS2 expression as a critical negative regulator of sustained anti-cancer efficacy of the PD-1 blockade that operates at the level of both tumor cells and leukocytes.

Mechanisms of resistance to proficient adaptive immune responses are being progressively dissected. Minn’s group reported that both type I and II IFN maintain the resistance program to ICB-induced adaptive immunity in a PD-L1-independent fashion.^47^ Indeed, both type I and II IFN signaling allowed tumors to acquire STAT1-related epigenomic changes, promoting the coordinated expression of IFN-stimulated genes and ligands for multiple T cell inhibitory receptors. Knockdown of both IFN receptors on tumor cells considerably improved the response to the combination of radiotherapy with anti-CTLA-4 mAb through specific down regulation of genes associated with acquired resistance. Therefore, PD-1^high^ Eomes^high^ T cells expressing the whole panel of inhibitory receptors became exhausted upon engagement with IFN-induced ligands harbored by tumor cells, unless interventions on the tumor cell bottlenecks JAK1/JAK2 down-regulated the cascade culminating in surface expression of inhibitory ligands on tumor cells.^47^ *Ifit* and *Mx1*, when considered as a two-feature metagene, together with non-synonymous single nucleotide variant load, were the most relevant genomic features associated with resistance to PD-1 blockade in 27 melanoma patients. Hence, our preclinical study and our study of a clinical cohort of melanoma patients confirms the crucial role of cell autonomous type I IFN receptor signaling, IFNAR1, in the progressive loss of sustained efficacy of PD-1 blockade, and resistance to combination ICB, revealing a new mechanism for this puzzling resistance program.

Admittedly, such findings are at odds with previous reports showing that early production of type I IFNs promotes DC activation and T cell cross-priming and that IFNAR signaling on host and tumor cells is crucial early during the effector phase, to ensure optimal antigen processing and minimal MHC-class I expression. In addition, two case reports described melanoma patients who initially responded to anti-PD-1 mAb but exhibited a late relapse attributed to loss-of-function mutations in *JAK1* or *JAK2*.^26^ In parallel, another team reported melanoma patients who failed to respond to anti-CTLA-4 mAb whose tumors harbored copy number alterations in IFNγ pathway genes.^23^ Together these data suggest an emerging framework for resistance to ICB that consists of primary resistance, a Darwinian process culminating in the emergence of distinct tumor clones with selective and intrinsic growth/survival advantage (i.e.; “cold” tumors, MHC deficiencies, lack of relevant tumor antigens, PD-L1 expression without TILs, “TGFβ-like” transcriptional signature with concomitant up-regulation of the expression of genes involved in mesenchymal transition, cell adhesion, extracellular matrix remodeling, angiogenesis, and wound healing),^48^ or acquired resistance, independent from PD-L1 expression, as a result of direct effector T cell-mediated selective pressure. Both may be related to mutational or genetic events. The copy number loss in IFN pathway genes (such as *Ifngr1*, *Irf1*, *Jak2*, and *Ifngr2*), or the amplification of crucial IFNγ pathway inhibitors, including SOCS1 and PIAS4 and loss-of-function mutations in *JAK1*/*JAK2* represent paradigms of primary and/or acquired resistance mechanisms to ICB. From a teleological point of view, such genetic events may be selected to circumvent the cytostatic and/or cytotoxic effects of IFNs.^23,26^ However, in contrast to loss-of-function resistance mechanisms, adaptive resistance could be viewed as a negative feedback loop dulling anti-tumor T cell activity through functional IFN receptor signaling pathways, resulting in the upregulation of immunosuppressive PD-L1 or galectins^47^ or NOS2 as exemplified here. Interestingly, these theories extend not only to ICB or immune-based therapies but also to targeted therapeutic strategies. Genomic and transcriptomic features modulating the response to MAPK inhibitors (MAPKi) are being described in melanoma to account for the primary or secondary resistance. The genetic variants positively selected by MAPKi were not highly recurrent and could not fully explain clinical relapse.^49^ Instead, gene signature-based transcriptomic alterations in acquired MAPKi-resistant melanoma were highly recurrent, encompassing not only cell autonomous pathways (*c-MET*, *LEF1*, *YAP1*) but also patterns of CD8^+^ T-cell exhaustion such as down-regulation of antigen presentation machinery, dominance of M2 macrophages and NF-kB activation in the TME.^48,49^

The production of nitric oxide (NO) in cells results from the conversion of L-arginine to L-citrulline by the NOS enzymes. NO regulates neurotransmission, immune responses and antimicrobial responses. Its role during the various stages of oncogenesis has also been well exemplified. NO acts at cell autonomous levels such as DNA damage, oncogene activation, inhibition of DNA repair enzymes, and tumor suppressor genes, modulation of apoptosis and metastases.^50^ The anti-tumor effects of NO produced by the immune defense were exemplified in various human tumors, while the pro-tumorigenic and immunosuppressive effects of NO (produced by M2 macrophages, MDSC, tumor or endothelial cells and neutrophils) were demonstrated in progressing tumors and metastases.^50^ Therapy-induced NO can also translocate and increase aggressiveness of non-targeted bystander cells.^51^ NO mediates the nitration of tyrosine residues in multiple proteins, thereby lessening the Th1 gene signature in M1 macrophages.^52^

In our study, *NOS2* was 100 to 1000 times more highly expressed than other NOS isoforms in the hematopoietic and cancer cell compartments. Therefore, the major source of NO in the tumors is due to the enzymatic activity of NOS2. The regulation of its expression in tumors has been previously widely studied. At the genetic and epigenetic level, polymorphisms in the *Nos2* gene or DNA methylation in the *Nos2* promotor influence activation or silencing of its enzymatic activity in tissues.^50^ Regarding non-genetic regulatory mechanisms, *Nos2* is primarily regulated at the expression level by inflammatory cytokines (TNFα, IL-1β, IL-6 and IFNγ), lipopolysaccharide, hypoxia, oxidative stress and HSP70 (ref. ^50^). One report showed that *Nos2* can be dependent on type I IFN signaling, especially STAT1, STAT2, Irf3 and NF-κB, involving pattern recognition receptors.^53^ Hence, our observation that NOS2 can be regulated in the TME by type I IFNs is original and adds to the complexity of NOS2 regulation. Supporting this notion, type I IFN-induced NOS2 in macrophages reduced intracellular accumulation of *Leishmania major*^54,55^ while favoring infection with *Mycobacterium tuberculosis*.^56^ IFNγ-induced NOS2 was also shown to reduce CD4^+^ T cell expansion in vitro following T cell activation with anti-PD-L1 or PD-1 mAbs, and NOS2 inhibitors were able to restore CD4^+^ T cell proliferation.^57^

Our results indicate that the overexpression of Nos2 in the context of PD-1 inhibition may occur early during treatment and, importantly, may precede resistance to anti-PD-1 therapy. Both the tumor and leukocyte cell fractions upregulate Nos2, the latter being mostly represented by CD11c^+^IAIE^hi^ cells, with DC being the most prominent producers in vitro. Given that L-NAME failed to improve the antitumor efficacy of the anti-PD-1 mAb in *Nos2*-deficient mice, and that L-NAME had some activity against distinct clones of *Ifnar1*-deficient sarcomas, we postulate that the functionally relevant source of NO is likely antigen presenting cells. The main mode of action of L-NAME was its capacity to activate DC and to reduce Treg accumulation in tumor beds, thereby increasing the CD8^+^/FOXP3 ratio in the context of a long-term PD-1 blockade. Therefore, our study supports the development of strategies aimed at restraining NOS2 activity and/or NO release to counteract PD-L1-independent resistance pathways elicited during PD-1 blockade.

## Material and methods

### Mouse studies

All experiments were approved by the local institutional boards. Experiments were performed in accordance with government and institutional guidelines and regulations. *Ifnar1*^−/−^ mice were originally kindly provided by the Professor Gilles Uzé (Université de Montpellier II, Montpellier, France) and the strain was maintained in the animal facility of Gustave Roussy Cancer Campus. *Nos2*^*−/−*^ mice were kindly provided by either the Doctor Armelle Prevost-Blondel (Institut Cochin, Paris, France) or by the Professor Vincenzo Bronte (Verona University Hospital, Verona, Italy). *Arg1*^*fl/fl*^*Tie2*^*Cre/+*^ and *Arg1*^*fl/fl*^ mice were kindly provided by the Professor Vincenzo Bronte (Istituto Oncologico Veneto, Padova, Italy).^58^ Females and males C57BL/6 were purchased from Harlan (France). Only females were used in experiments with the exception of specific KO strains and their respective controls. Mice were used between 8 and 16 weeks of age. All mice experiments were performed at Gustave Roussy Cancer Campus, France or at the Istituto Oncologico Veneto, Italy and mice were housed in specific pathogen-free conditions in both animal facilities or were maintained in isolators.

### Mouse cell lines

The AT3 cell line was kindly provided by the Professor Mark Smyth (QIMR Berghofer Medical Research Institute, Brisbane, Australia). MCA205 derived IFNAR1 and PD-L1 KO clones were generated as described below. The MCA205WT and MCA205OVA sarcoma, MC38 carcinoma and AT3 mammary carcinoma cell lines were cultured at 37 °C under 5% CO_2_ in RPMI-1640 medium supplemented with 10% heat-inactivated fetal bovine serum (FBS), 1% penicillin/streptomycin, 2 mM l-glutamine and 1% of sodium pyruvate and non-essential amino acids (all from Gibco-Invitrogen), referred herein as complete RPMI medium. The right flank of mice was subcutaneously (*s.c.*) injected with 0.8 × 10^6^ cells for MCA205WT and 1 × 10^6^ cells for MCA205OVA, MC38 and AT3. Mouse cell lines were regularly tested for mycoplasma contamination and cells were not used for more than 10 passages.

### CRISPR/Cas9 and Zinc Finger clones

*Ifnar1*^−/−^ MCA205 cell lines were generated by means of the CompoZr^®^ Zinc Finger Nuclease Technology (Sigma-Aldrich), as per manufacturer’s recommendations as previously reported.^38,59^ *Pdl1*^−/−^ MCA205 cell lines were generated using the CRISPR/Cas9 technology (pCMV-Cas9-GFP, Sigma-Aldrich), as per manufacturers’ protocols. After transfection, single GFP positive cells were sorted in 96 well-plates for the establishment of different clones. After the generation of clones, IFNAR1 and PD-L1 expressions were checked by flow cytometry and functional assays.

### Bone marrow derived-DC and myeloid cell cultures

Bone marrow-derived dendritic cells (BMDCs) and bone marrow-derived myeloid cells (BMMCs) were generated using femurs and tibias of females C57BL/6 WT and *Ifnar1*^*−/−*^ mice aged of 8 to 12 weeks. Bones were carefully collected in sterile PBS. After washing bones in alcohol and Iscove’s medium (IMDM, Sigma-Aldrich) baths, extremities of bones were cut and flushed using a 26 G needle. After red blood cell lysis with ACK buffer, cells were cultured in IMDM supplemented with 10% of FCS + 2 mM l-Glutamine + 100 UI/mL Penicillin/Streptomycin + 50 µM 2-mercaptoethanol (Sigma-Aldrich) (referred herein as complete IMDM medium) at 0.5 × 10^6^/mL and treated with 10 ng/mL of GM-CSF and IL-4 for BMDCs and 50 ng/mL of M-CSF for BMMCs (all from Peprotech). Cells were split at day 3 and used at day 7 or 8.

### Therapies

#### In vitro stimulations

Tumor cell lines were cultured in complete RPMI medium at 0.5 × 10^6^/mL in 48 well plates in triplicates and treated with IFNα (from Miltenyi Biotech) or IFNγ (eBioscience) at 1000 IU/mL. BMDCs or BMMCs cells were cultured in complete IMDM at 0.5 × 10^6^/mL in 48 well-plates in triplicates and treated with 1000 IU/mL of IFNα (from Miltenyi Biotech, Germany) or 100 ng/mL of ultrapure LPS (InvivoGen). After 24 h of incubation, supernatants were collected and stored at –80 °C until cytokine measurements. In parallel, cells were harvested, washed and pre-incubated with anti-CD16/32 Ab (clone 93, eBioscience) for 20 min at 4 °C and then stained to discriminate populations and markers of interest using the antibodies listed in Supplementary information, Table S4. In vivo *treatments*. Mice were treated intraperitoneally (i.p.) when tumors became palpable (20 to 25 mm²) with anti-PD-1 mAb (250 µg/mouse; clone RMP1–14, BioXcell, NH, USA), with or without anti-CD80 (500 µg/mouse; clone 1G10, BioXcell, NH, USA), anti-PD-L1 (250 µg/mouse; clone 10 F.9G2, BioXcell, NH, USA), anti-CTLA-4 (100 µg/mouse; clone 9D9, BioXcell, NH, USA) (or the appropriate isotype control) mAb as described in the figures or figure legends. Early or late injections correspond to one day prior (anti-CD80 early) or the same day (anti-PD-L1 early) as the first anti-PD-1 infusions or one day prior (anti-CD80 late) or concomitantly (anti-PD-L1 late) with the third anti-PD-1 treatment, respectively, for four injections every three days. Abatacept was kindly given by Professor Antoine Durrbach (Hôpital Bicêtre, Le Kremlin-Bicêtre, France) and dissolved in sterile PBS prior to i.p. injections in mice at 10 mg/kg. Inhibition of NOS was performed using L-NAME dissolved in drinking water, given ad libitum, at 1 g/L started one day prior anti-PD-1 Ab-based treatment until the end of the experiment. Bottles were changed every 2 to 3 days.

### Flow cytometry analyses and cell-sorting

Mice were sacrificed 2–3 days after the final anti-PD-1 or isotype control antibody treatments to assess immune parameters in the spleen and in the tumor bed. Briefly, tumors were cut into small pieces and digested in RPMI medium containing Liberase^TM^ at 25 μg/mL (Roche) and DNase1 at 150 IU/mL (Roche) for 30 min at 37 °C. Cells were then filtered through a 100-µm cell strainer, and splenocytes at a 2 × 10^6^/mL concentration (after red blood cell lysis) or TME-derived cells were preincubated with purified anti-mouse CD16/32 mAb (clone 93, eBioscience) for 20 min at 4 °C before membrane staining. Dead cells were excluded using the live/dead fixable yellow dead cell stain kit (Life Technologies^TM^). For membrane staining, antibodies were incubated 20 min at 4 °C to discriminate populations of interest. To assess intracellular cytokine production, 2 × 10^6^ cells were stimulated with PMA (50 ng/mL) and ionomycine (1 μg/mL) (all from Sigma Aldrich) in the presence of Golgi Stop (BD Biosciences). After 4 h of stimulation at 37 °C, cells were membrane stained and then permeabilized using BD Cytofix/Cytoperm Kit (BD Biosciences) during 20 min at 4 °C, washed and intracellularly stained during 30 min at 4 °C with anti-TNFα, anti-IFNγ and anti-IL-17A Abs. For Nos2 and Arg1 protein level expressions, cells were membrane stained, permeabilized using BD Cytofix/Cytoperm Kit (BD Biosciences) during 20 min at 4 °C, washed and then intracellularly stained during 30 min at 4 °C with anti-Nos2 and anti-Arg1 Abs. To assess cell proliferation and proportion of regulatory CD4^+^ T cells after surface staining, cells were permeabilized during 45 min at 4 °C using the Foxp3 kit (eBioscience) and then washed and stained with anti-Ki67 and anti-Foxp3 Abs during 30 min at 4 °C. Antibodies are detailed in the Supplementary information, Table S4. Acquisition was performed on a Cyan ADP 9 Color Flow Cytometer (Beckman Coulter) after appropriate compensation using mono-stained cells. Data were analyzed with FlowJo software (Tree Star), version 7.6.5. To isolate cells following tumor digestion, the digested tissue was incubated in Fc-block and then stained during 20 min at 4 °C with anti-CD45.2 Ab in Automacs Buffer (Miltenyi Biotech). Cell sorting was carried out on either a BD Influx or a BD Aria III Flow cytometer. Cells were immediately resuspended in RLT^+^ Buffer and stored at –80 °C until RNA extraction.

### Gene expression analyses

#### Culture conditions

Tumor cell lines and BMMCs/BMDCs were cultured at 0.02 × 10^6^/mL in 96 well flat bottom-plates (Nunc MaxiSorp, sterilized during 30 min under UV) in complete medium and treated either with IFNα (from Miltenyi Biotech), IFNγ (eBioscience), both at 1000 IU/mL or 100 ng/mL of ultrapure LPS (InvivoGen). 24 h later, supernatant was collected after centrifugation and 12 wells were pooled together in RLT^+^ buffer to form one replicate and kept for gene expression analyses. *RNA extraction*. Total RNA extraction and genomic DNA removal were performed with the RNeasy Mini kit (Qiagen), following the manufacturer’s recommendations. *Reverse transcription*. A maximum of 1 µg of RNA, measured by using a NanoDrop^TM^ Spectrophotometer (Thermo Fischer Scientific), was reverse transcribed into cDNA with a mix composed of SuperScript III Reverse Transcriptase (Life Technologies), RNaseOUT^TM^ Recombinant Ribonuclease Inhibitor (Life Technologies), Random primers (Promega) and Deoxynucleoside Triphosphate Set, PCR grade (Roche Diagnostics). *Quantitative gene expression assay*. Expression of *β2* *m* (Mm00437762_m1), *Ppia* (Mm02342429_g1), *Ifnβ1* (Mm00439552_s1), *Nos2* (Mm00440502_m1), *Nos1* (Mm01208059_m1), *Nos3* (Mm00435217_m1), *Arg1* (Mm00475988_m1), *Isg15* (Mm01705338_s1), *Mx1* (Mm01218004_m1), and *Pdl1* (Mm0045054_m1) (all from Life Technologies) was analyzed with the TaqMan® Gene Expression Assay using the Universal Master Mix II on a StepOnePlus™ Real-Time PCR System (Life Technologies). Amplifications were carried out using the following ramping profile: 1 cycle at 95 °C for 10 min, followed by 45 cycles of 95 °C for 30 s, 60 °C for 1 min. Quantitative RT-PCR data were normalized to the expression levels of the housekeeping genes *β2* *m* or *Ppia*, as indicated in each figure, by means of the 2^−ΔCt^ method multiplied by 10^6^.

### Cytokine quantification

CXCL10 was measured by ELISA (BD Biosciences) according to the manufacturer’s recommendations.

### Microarray analysis

The CD45^+^ fraction from MC38 tumors was subjected to cell-sorting and extracted as described above. RNA was subjected to control quality using a Bioanalyzer 2100. GeneChip Mouse Gene 2.0ST arrays (Affymetrix) were used to analyze the gene expression profile of CD45^+^ samples. This was performed at the Genomics Platform of the Cochin Institute according to standard validated protocols. Data analyses and representations were performed with the R software (http://www.R-project.org/). To assess the statistical significance of the differential gene expression, the empirical Bayes statistics for linear model (series of probeset arrays) was used (limma package).^60^ Selection criteria were as follows: |logFC| > log_2_(1.5), *p*-value < 0.05. Heatmap representations are normalized by row. The raw data are available upon request to the lead author.

### Human studies

All patients provided informed consent before enrollment in these studies. In vitro *stimulations*. Peripheral blood mononuclear cells (PBMCs) were isolated from blood of healthy volunteers using a Ficoll-Hypaque density gradient media (PAA Laboratories). After centrifugation, PBMCs were collected, washed, counted and then used for in vitro experiments. Tumor infiltrated leukocytes (TILs) were isolated from metastatic lesions of melanoma patients as previously described.^61^ Briefly, tumor samples were minced and then digested using a mix of Collagenase IV (50 IU/mL), hyaluronidase (280 IU/mL) and Dnase1 (30 IU/mL) (all from Sigma-Aldrich) in RPMI1640 + 1% penicillin/streptomycin (Gibco Inviotrogen). Samples were subjected to gentleMACS dissociation (Miltenyi Biotech). Digested samples were washed in PBS and passed through a 70 μm cell strainer before to be counted and stored in liquid nitrogen using CryoMaxx medium (PAA Laboratories).^61^ PBMCs and TILs were quickly thawed in culture media (RPMI1640 [Gibco, Invitrogen] + 10% human AB^+^ serum [Institut de Biotechnologies Jacques Boy] + 2 mM l-Glutamine + 1% sodium/pyruvate + 1% penicillin/streptomycin [all from Gibco, Invitrogen]), washed, counted and seeded at 0.3 × 10^6^/mL per well in 48 well-plate in culture medium and incubated with or without Roferon® (1000 UI/mL, Roche Pharma) and LPS (10 ng/mL, Sigma). After 24–60 h of incubation, cells were harvested, washed, immediately resuspended in RLT + Buffer and stored at –80 °C until RNA extraction and quantification.

### Gene expression analyses

Total RNA extraction and genomic DNA removal were performed with the RNeasy Mini kit (Qiagen), following manufacturer’s recommendations and quantified using a NanoDrop^TM^ Spectrophotometer (Thermo Fisher Scientific). RNA was either reverse transcribed into cDNA or RT-PCR amplifications were performed using TaqMan™ RNA-to-CT™ 1-Step Kit (ThermoFisher Scientific) *Reverse transcription*. A maximum of 1 µg of RNA was reverse transcribed into cDNA with a mix comprising SuperScript III Reverse Transcriptase (Life Technologies), RNaseOUT^TM^ Recombinant Ribonuclease Inhibitor (Life Technologies), Random primers (Promega) and Deoxynucleoside Triphosphate Set, PCR grade (Roche Diagnostics). *Quantitative gene expression assay*. Expression of *β2* *M* (Hs00187842_m1), *NOS2* (Hs01075529_m1), *NOS3* (Hs01574665_m1) and *PDL1* (Hs00204257_m1) (all from Life Technologies) were analyzed with TaqMan® Gene Expression Assay using the Universal Master Mix II. Amplifications were carried out using the following ramping profile: 1 cycle at 95 °C for 10 min, followed by 45 cycles of 95 °C for 30 s, 60 °C for 1 min. *1-Step qRT-PCR*. A maximum of 500 ng of RNA was used in 25 µL reaction volumes, using the same ramping profile as above with the addition of 1 cycle at 48 °C for 15 min at the beginning of the amplification. All reactions were perfomed on a StepOnePlus™ or QuantStudio 3 Real-Time PCR System (Life Technologies). Quantitative RT-PCR data were normalized to the expression levels of the housekeeping genes *β2M* by means of the 2^−ΔΔCt^, taking into account the values obtained from the control conditions.

### Biospecimen collection at the MD Anderson

(Andrews M.C. et al, manuscript in preparation) Wargo’s team assembled a cohort of patients with metastatic melanoma receiving combination of ICB either on clinical trials or as standard of care (SOC) therapy between 01/01/2014 and 08/31/2017. Patients were excluded if insufficient data were available to determine radiographic responses. Patients were classified as “responders” (R, being complete response + partial response) or “non-responders” (NR, being stable disease + progressive disease) based on their best overall response (BOR) to ICBs measured by RECIST v1.1. This cohort comprised patients with stage IV disease (*n* = 22, 96%) or unresectable stage IIIC (*n* = 1, 4%) and similar numbers of systemic therapy-naïve or pre-treated patients (*n* = 12 (52%) vs. 11 (48%), respectively); notably, one third (*n* = 7, 30%) of patients had received prior pharmacologic immune-stimulating therapies (*n* = 3 IFN, *n* = 3 IL-2, *n* = 3 ipilimumab monotherapy, *n* = 3 pembrolizumab monotherapy, *n* = 1 atezolizumab monotherapy). Available pre-treatment tumor samples were identified and retrieved for correlative molecular analyses. Gene expression profiling was performed using a custom-designed 795-gene code-set as previously described (Supplementary information, Table S3).^62^ Within-cohort housekeeping genes were selected by identifying the two most stably-expressed genes based on genewise CV within each of the upper, lower, and interquartile ranges of genes (lower: IFIT1B, IL20; interquartile: IKBKG, TRIM5; upper: ACTB, ZC3HAV1). Differential gene expression analyses were performed on count data using the R package NanoStringDiff (v1.10.0).^63^ For statistical significance, fold change of >2 or < 0.5 (calculated as the ratio of average gene expression intensity in R versus NR) with an FDR adjusted *p* < 0.10 was used. Normalized data (for visualizations) were generated from raw count data using the variance stabilizing normalization implemented in the R package NanoStringNorm (v1.2.1).^64^

### Human tumor histology

#### Immunohistochemistry, Scanning and Analyzing of Systems

Composition and cellularity were assessed using a hematoxylin, eosin & safran stain (H&E). The staining of CD68 and NOS2 were performed on an automated immunostainer (The BenchMark ULTRA, Ventana, Gustave Roussy). Heat-induced antigen retrieval in EDTA buffer (pH 8.0) was performed respectively for 32 min (CD68) and for 64 min (NOS2) at 95 °C. The mouse monoclonal anti-human CD68 antibody (clone PG-M1, 30 mg/mL, #M0876, Dako Agilent) was diluted 1:200 in antibody diluent (Zytomed) and the slides were incubated for 1 h at room temperature. The mouse monoclonal anti-human NOS2 antibody (4.8 mg/mL, #DMAB5712RH, Diagnostic Creative) was diluted 1:240 in antibody diluent (Zytomed) and the slides were incubated for 1 h at 37 °C. The biotin-free alkaline phosphatase system of detection technique with Fast red as chromogen was applied (ultraView Universal Alkaline Phosphatase Red Detection Kit, Ventana). The slides were also counterstained by Hematoxylin kit (Ventana). Images displayed in the figures were taken with Zeiss Axio Scan.Z2 (primary objective_20/0.4, ocular_objective_10) and exported from the Zen 2 lite software as TIFF images. Some images were processed with using algorithm developed in Visiopharm Integrator System (VIS) (Visiopharm A/S, Denmark). WSI with the stains H&E, CD68 and NOS2 were saved and stacked thanks to the Visiopharm module TISSUEalign. Intratumoral and peritumoral regions were then defined manually on the H&E layer. After CD68 positive cells were identified and counted in those regions, a cell density map (heatmap) was computed and regions with the highest density of CD68 positive cells (hot spots) were automatically identified. Then the density of NOS2 positive surface was quantified on these hotspots of CD68 positive cells. 4 patients out of 11 were not able to be quantified and, therefore, were excluded from the final analysis represented in Supplementary information, Fig. S10. Patient’s characteristics of the total cohort can be found in Supplementary information, Table S2.

### Statistical analyses

Data analyses and representations were performed either with the R software (http://www.R-project.org/) or Prism 5 (GraphPad, San Diego, CA, USA). Statistical analyses gathering more than two groups were performed using ANOVA followed with pairwise comparisons with Bonferroni adjustments. Otherwise, for two groups, statistical analyses were performed using the unpaired *t*-test or Wilcoxon matched-pairs signed rank test. Outliers within a given distribution were tested using Grubbs’ test (https://graphpad.com/quickcalcs/Grubbs1.cfm) with a threshold at *p* < 0.05. Tumor growth experiments were analyzed with the TumGrowth software (https://kroemerlab.shinyapps.io/TumGrowth/),^65^ with default settings at the exception of the original tumor measurements that were log transformed before linear mixed-effect modeling. Cox proportional hazards modelling were applied when assessing the impact of the treatment on mice survival. Unless stated, all *p*-values are reported after Bonferroni correction when the question is addressing more than 2 experimental conditions. *p*-values were two-sided with 95% confidence intervals and were considered significant when *p* < 0.05. Symbol significance: *p < 0.05, **p < 0.01, ***p < 0.001.

## Supplementary information

Supplementary information, Fig S1. Sensitivity and resistance to PD-1 therapy in various tumor models

Supplementary information, Fig S2. Surrogate immune hallmarks of response to PD-1 blockade

Supplementary information, Fig S3. IFN-induced tumor cell expression of MHC class I, IFNAR1 and PD-L1 molecules and secretion of CXCL10 in vitro

Supplementary information, Fig S4. IFNβ and ISG gene expression levels in CD45- cells isolated from MCA205WT and MC38 tumors

Supplementary information, Fig S5. Global PD-L1 expression upregulation during PD-1 blockade and post IFN stimulation

Supplementary information, Fig S6. CD80/86 engagement or tumoral PD-L1 expression are not associated with resistance to anti-PD-1 therapy

Supplementary information, Fig S7. Flow cytometric analyses of ARG1 and NOS2 in BMDCs and BMMCs

Supplementary information, Fig S8. Expressions of Nos1 and Nos3 are not modulated in the TME during the course of anti-PD-1 mAbs

Supplementary information, Fig S9. Therapeutic resistance to anti-PD-1 mAb is Arginase 1 independent

Supplementary information, Fig S10. NOS2 upregulation post anti-PD-1 therapy in melanoma patients

Supplementary information, Fig S11

Table S1 : Genes list found differentially expressed post anti-PD-1 treatment in CD45+ cells

Table S2. Related to Figure S8 (B-C). Patient ’s characteristics

Table S3: Details of the custom-designed 795-gene codeset

Table S4: Antibodies

## References

[1] Hanahan D, Weinberg RA (2011). Hallmarks of cancer: the next generation. Cell.

[2] Smyth MJ, Ngiow SF, Ribas A, Teng MW (2016). Combination cancer immunotherapies tailored to the tumour microenvironment. Nat. Rev. Clin. Oncol..

[3] Zitvogel L, Tesniere A, Kroemer G (2006). Cancer despite immunosurveillance: immunoselection and immunosubversion. Nat. Rev. Immunol..

[4] Le DT (2015). PD-1 blockade in tumors with mismatch-repair deficiency. N. Engl. J. Med..

[5] Riaz N (2016). Recurrent SERPINB3 and SERPINB4 mutations in patients who respond to anti-CTLA4 immunotherapy. Nat. Genet..

[6] Rizvi NA (2015). Cancer immunology. Mutational landscape determines sensitivity to PD-1 blockade in non-small cell lung cancer. Science.

[7] Schumacher TN, Schreiber RD (2015). Neoantigens in cancer immunotherapy. Science.

[8] Van Allen EM (2015). Genomic correlates of response to CTLA-4 blockade in metastatic melanoma. Science.

[9] Sharma P, Allison JP (2015). Immune checkpoint targeting in cancer therapy: toward combination strategies with curative potential. Cell.

[10] Sharma P, Allison JP (2015). The future of immune checkpoint therapy. Science.

[11] Restifo NP, Dudley ME, Rosenberg SA (2012). Adoptive immunotherapy for cancer: harnessing the T cell response. Nat. Rev. Immunol..

[12] Borghaei H (2015). Nivolumab versus docetaxel in advanced nonsquamous non-small-cell lung cancer. N. Engl. J. Med..

[13] Nghiem PT (2016). PD-1 Blockade with pembrolizumab in advanced merkel-cell carcinoma. N. Engl. J. Med..

[14] Robert C (2011). Ipilimumab plus dacarbazine for previously untreated metastatic melanoma. N. Engl. J. Med..

[15] Rosenberg JE (2016). Atezolizumab in patients with locally advanced and metastatic urothelial carcinoma who have progressed following treatment with platinum-based chemotherapy: a single-arm, multicentre, phase 2 trial. Lancet.

[16] Hirsch, L., Zitvogel, L., Eggermont, A. & Marabelle, A. PD-Loma: a cancer entity with a shared sensitivity to the PD-1/PD-L1 pathway blockade. *Br J Cancer.* **120**, 3–5 (2019). 10.1038/s41416-018-0294-4 PMC6325162 30413824

[17] Hargadon KM, Johnson CE, Williams CJ (2018). Immune checkpoint blockade therapy for cancer: an overview of FDA-approved immune checkpoint inhibitors. Int. Immunopharmacol..

[18] Pardoll D (2015). Cancer and the immune system: basic concepts and targets for intervention. Semin. Oncol..

[19] Topalian SL (2012). Safety, activity, and immune correlates of anti-PD-1 antibody in cancer. N. Engl. J. Med..

[20] Gandhi L (2018). Pembrolizumab plus chemotherapy in metastatic non-small-cell lung cancer. N. Engl. J. Med..

[21] Spranger S, Bao R, Gajewski TF (2015). Melanoma-intrinsic beta-catenin signalling prevents anti-tumour immunity. Nature.

[22] Neubert NJ (2018). T cell-induced CSF1 promotes melanoma resistance to PD1 blockade. Sci. Transl. Med..

[23] Gao J (2016). Loss of IFN-gamma pathway genes in tumor cells as a mechanism of resistance to anti-ctla-4 therapy. Cell.

[24] Koyama S (2016). Adaptive resistance to therapeutic PD-1 blockade is associated with upregulation of alternative immune checkpoints. Nat. Commun..

[25] Ribas A (2016). Association of pembrolizumab with tumor response and survival among patients with advanced melanoma. JAMA.

[26] Zaretsky JM (2016). Mutations associated with acquired resistance to pd-1 blockade in melanoma. N. Engl. J. Med..

[27] Sade-Feldman M (2017). Resistance to checkpoint blockade therapy through inactivation of antigen presentation. Nat. Commun..

[28] Khong HT, Restifo NP (2002). Natural selection of tumor variants in the generation of “tumor escape” phenotypes. Nat. Immunol..

[29] Restifo NP (1996). Loss of functional beta 2-microglobulin in metastatic melanomas from five patients receiving immunotherapy. J. Natl. Cancer Inst..

[30] Pardoll DM (2012). The blockade of immune checkpoints in cancer immunotherapy. Nat. Rev. Cancer.

[31] Taube JM (2012). Colocalization of inflammatory response with B7-h1 expression in human melanocytic lesions supports an adaptive resistance mechanism of immune escape. Sci. Transl. Med..

[32] Landsberg J (2012). Melanomas resist T-cell therapy through inflammation-induced reversible dedifferentiation. Nature.

[33] Ngiow SF (2015). A threshold level of intratumor CD8+ T-cell PD1 expression dictates therapeutic response to Anti-PD1. Cancer Res..

[34] Gajewski TF, Corrales L (2015). New perspectives on type I IFNs in cancer. Cytokine Growth Factor Rev..

[35] Rodriguez-Ruiz ME (2016). Abscopal effects of radiotherapy are enhanced by combined immunostimulatory mabs and are dependent on cd8 t cells and crosspriming. Cancer Res..

[36] Smyth MJ, Dunn GP, Schreiber RD (2006). Cancer immunosurveillance and immunoediting: the roles of immunity in suppressing tumor development and shaping tumor immunogenicity. Adv. Immunol..

[37] Wang X (2017). Suppression of type i ifn signaling in tumors mediates resistance to anti-pd-1 treatment that can be overcome by radiotherapy. Cancer Res..

[38] Sistigu A (2014). Cancer cell-autonomous contribution of type I interferon signaling to the efficacy of chemotherapy. Nat. Med..

[39] Dunn GP (2005). A critical function for type I interferons in cancer immunoediting. Nat. Immunol..

[40] Bronte V (2003). IL-4-induced arginase 1 suppresses alloreactive T cells in tumor-bearing mice. J. Immunol..

[41] Dussurget O, Bierne H, Cossart P (2014). The bacterial pathogen Listeria monocytogenes and the interferon family: type I, type II and type III interferons. Front. Cell. Infect. Microbiol..

[42] Mondanelli G, Ugel S, Grohmann U, Bronte V (2017). The immune regulation in cancer by the amino acid metabolizing enzymes ARG and IDO. Curr. Opin. Pharm..

[43] Rodriguez PC (2004). Arginase I production in the tumor microenvironment by mature myeloid cells inhibits T-cell receptor expression and antigen-specific T-cell responses. Cancer Res..

[44] Rodriguez PC, Ochoa AC (2008). Arginine regulation by myeloid derived suppressor cells and tolerance in cancer: mechanisms and therapeutic perspectives. Immunol. Rev..

[45] Modolell M, Corraliza IM, Link F, Soler G, Eichmann K (1995). Reciprocal regulation of the nitric oxide synthase/arginase balance in mouse bone marrow-derived macrophages by TH1 and TH2 cytokines. Eur. J. Immunol..

[46] Verbeke H (2011). Isotypic neutralizing antibodies against mouse GCP-2/CXCL6 inhibit melanoma growth and metastasis. Cancer Lett..

[47] Benci JL (2016). Tumor interferon signaling regulates a multigenic resistance program to immune checkpoint blockade. Cell.

[48] Hugo W (2016). Genomic and transcriptomic features of response to anti-PD-1 therapy in metastatic melanoma. Cell.

[49] Hugo W (2015). Non-genomic and immune evolution of melanoma acquiring MAPKi resistance. Cell.

[50] Vannini F, Kashfi K, Nath N (2015). The dual role of iNOS in cancer. Redox Biol..

[51] Girotti AW, Fahey JM, Korytowski W (2016). Multiple means by which nitric oxide can antagonize photodynamic therapy. Curr. Med. Chem..

[52] Lu G (2015). Myeloid cell-derived inducible nitric oxide synthase suppresses M1 macrophage polarization. Nat. Commun..

[53] Farlik M (2010). Nonconventional initiation complex assembly by STAT and NF-kappaB transcription factors regulates nitric oxide synthase expression. Immunity.

[54] Diefenbach A (1998). Type 1 interferon (IFNalpha/beta) and type 2 nitric oxide synthase regulate the innate immune response to a protozoan parasite. Immunity.

[55] Mattner J (2000). Regulation of type 2 nitric oxide synthase by type 1 interferons in macrophages infected with Leishmania major. Eur. J. Immunol..

[56] Moreira-Teixeira L (2016). Type I IFN inhibits alternative macrophage activation during mycobacterium tuberculosis infection and leads to enhanced protection in the absence of IFN-gamma signaling. J. Immunol..

[57] Yamazaki T (2005). Blockade of B7-H1 on macrophages suppresses CD4+ T cell proliferation by augmenting IFN-gamma-induced nitric oxide production. J. Immunol..

[58] Marigo I (2016). T cell cancer therapy requires CD40-CD40L activation of tumor necrosis factor and inducible nitric-oxide-synthase-producing dendritic cells. Cancer Cell.

[59] Gaj T, Guo J, Kato Y, Sirk SJ, Barbas CF (2012). Targeted gene knockout by direct delivery of zinc-finger nuclease proteins. Nat. Methods.

[60] Ritchie ME (2015). limma powers differential expression analyses for RNA-sequencing and microarray studies. Nucleic Acids Res..

[61] Jacquelot N (2016). Immunophenotyping of stage III melanoma reveals parameters associated with patient prognosis. J. Invest. Dermatol..

[62] Chen PL (2016). Analysis of immune signatures in longitudinal tumor samples yields insight into biomarkers of response and mechanisms of resistance to immune checkpoint blockade. Cancer Discov..

[63] Wang, H., Zhai, T. & Wang, C. NanoStringDiff: differential expression analysis of NanoString nCounter data. R package version 1.12.0 (2018).

[64] Waggott D (2012). NanoStringNorm: an extensible R package for the pre-processing of NanoString mRNA and miRNA data. Bioinformatics.

[65] Enot DP, Vacchelli E, Jacquelot N, Zitvogel L, Kroemer G (2018). TumGrowth: an open-access web tool for the statistical analysis of tumor growth curves. Oncoimmunology.

