# Supplementary material for: Sustained Type I interferon signaling as a mechanism of resistance to PD-1 blockade
Source: Cell Res. 2019 Sep 3;29(10):846–61. doi: 10.1038/s41422-019-0224-x (PMC6796942; doi:10.1038/s41422-019-0224-x)
Supplement: Supplementary file 1 — Supplementary information, Fig S1. Sensitivity and resistance to PD-1 therapy in various tumor models [file 41422_2019_224_MOESM1_ESM.pdf]

Figure S1

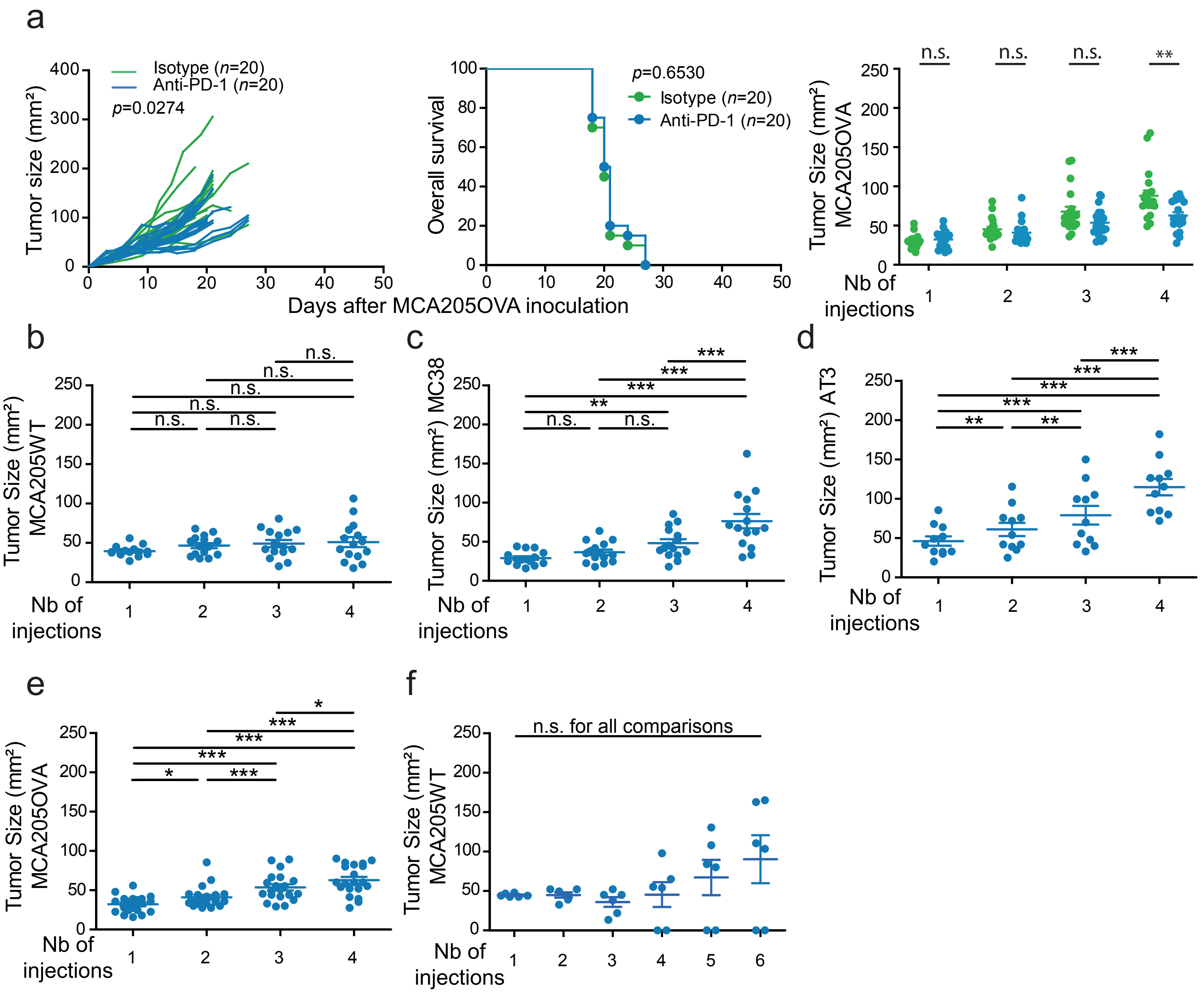
a
Isotype (n=20)
Anti-PD-1 (n=20)
p=0.0274
Tumor size (mm²)
p=0.6530
Overall survival
Days after MCA205OVA inoculation
n.s.
n.s.
n.s.
**
Tumor Size (mm²) MCA205OVA
Nb of injections
b
Tumor Size (mm²) MCA205WT
c
Tumor Size (mm²) MC38
d
Tumor Size (mm²) AT3
e
Tumor Size (mm²) MCA205OVA
f
n.s. for all comparisons
Tumor Size (mm²) MCA205WT
Nb of injections

Supplementary information, Fig S1. Sensitivity and resistance to PD-1 therapy in various tumor models.

(a) Tumor growth kinetics of MCA205OVA-expressing sarcoma (left panel), survival curves (middle panel) and tumor sizes after sequential injections of isotype or anti-PD-1 mAbs (right panel) are depicted. (b-f) Tumor sizes after sequential injections of anti-PD-1 mAb in MCA205WT (b and f), MC38 (c), AT3 (d) and MCA205OVA (e) tumor models are depicted. Each line or dot represents 1 mouse. The graphs depict pooled data from 1 (f), 2 (d), 3 (b and c) or 4 (a and e) independent experiments including 5-6 mice per group and per experiment. Statistical analyses were performed using unpaired t-tests (a, right panel) or ANOVA statistical tests and pairwise comparisons with Bonferroni adjustment (b-f). *$p<0.05$, **$p<0.01$, ***$p<0.001$, n.s.: not significant. Means ± SEM are represented.
