# Supplementary material for: Sustained Type I interferon signaling as a mechanism of resistance to PD-1 blockade
Source: Cell Res. 2019 Sep 3;29(10):846–61. doi: 10.1038/s41422-019-0224-x (PMC6796942; doi:10.1038/s41422-019-0224-x)
Supplement: Supplementary file 2 — Supplementary information, Fig S2. Surrogate immune hallmarks of response to PD-1 blockade [file 41422_2019_224_MOESM2_ESM.pdf]

Figure S2

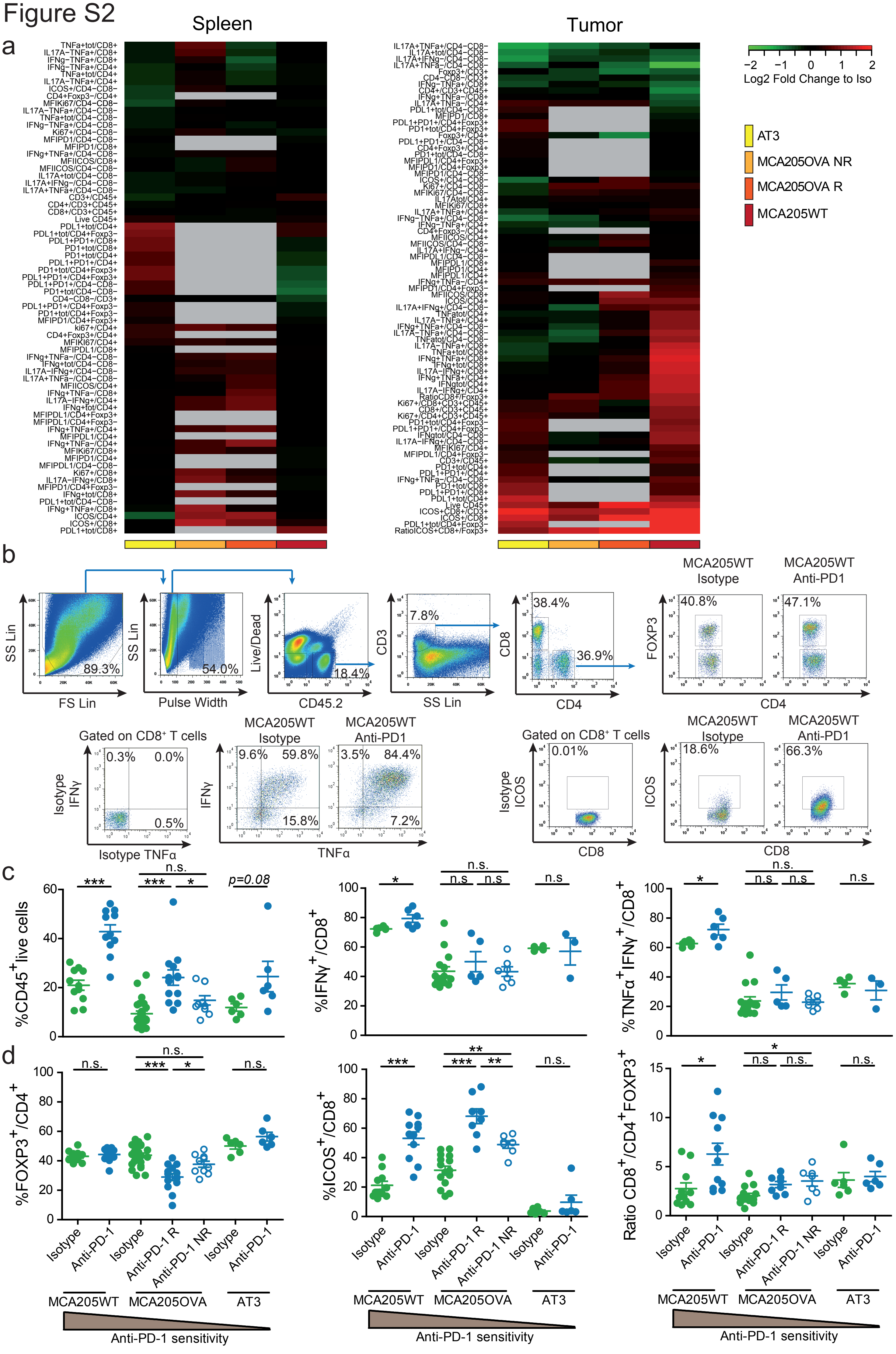
a
Spleen
Tumor
Log2 Fold Change to Iso
AT3
MCA205OVA NR
MCA205OVA R
MCA205WT
b
SS Lin
FS Lin
89.3%
Pulse Width
54.0%
Live/Dead
CD45.2
18.4%
CD3
SS Lin
7.8%
CD8
CD4
38.4%
36.9%
MCA205WT Isotype
MCA205WT Anti-PD1
FOXP3
40.8%
47.1%
Gated on CD8+ T cells
Isotype IFNγ
Isotype TNFα
0.3%
0.0%
0.5%
IFNγ
TNFα
9.6%
59.8%
15.8%
3.5%
84.4%
7.2%
Isotype ICOS
ICOS
CD8
0.01%
18.6%
66.3%
c
%CD45+live cells
%IFNγ+/CD8+
%TNFα+IFNγ+/CD8+
p=0.08
n.s.
d
%FOXP3+/CD4+
%ICOS+/CD8+
Ratio CD8+/CD4+FOXP3+
Isotype
Anti-PD-1
Anti-PD-1 R
Anti-PD-1 NR
MCA205WT
MCA205OVA
AT3
Anti-PD-1 sensitivity

Supplementary information, Fig S2. Surrogate immune hallmarks of response to PD-1 blockade.

(a) Spleens and tumors of mice bearing MCA205WT, MCA205OVA and AT3 were analyzed by flow cytometry 48 hrs after the fourth and last injections of anti-PD-1 or its isotype control mAbs. MCA205OVA tumors treated by anti-PD-1 were segregated into responders (R) and non-responders (NR) based on the tumor weight at sacrifice (relative to the isotype controls). Heat maps of the various immune parameters per tumor model based on the log_2 fold change relative to the isotype control. Gray cells represent parameters non-measured. (b-d) Most significant immunological changes in the tumor bed associated with responses to anti-PD-1 mAb. Representative gating strategy of MCA205WT tumor parameters (b). The percentages of $CD45^+$ cells (c, left panel), of $IFN\gamma^+$ and double positive $IFN\gamma TNF\alpha^+CD8$ T cells (c, middle and right panels, respectively), of Treg (d, left panel), of $CD8^+ICOS^+$ cells (d, middle panel) and the $CD8^+/CD4^+FOXP3^+$ ratio (d, right panel). These data were generated from 1-4 independent pooled experiments per tumor model and each dot represents 1 mouse. ANOVA statistical tests and pairwise comparisons with Bonferroni adjustment were used in (c) and (d) for MCA205OVA. Other statistical analyses were performed using unpaired t-tests. *$p<0.05$, **$p<0.01$, ***$p<0.001$, n.s.: not significant. Means $\pm$ SEM are represented.
