# Supplementary material for: Sustained Type I interferon signaling as a mechanism of resistance to PD-1 blockade
Source: Cell Res. 2019 Sep 3;29(10):846–61. doi: 10.1038/s41422-019-0224-x (PMC6796942; doi:10.1038/s41422-019-0224-x)
Supplement: Supplementary file 3 — Supplementary information, Fig S3. IFN-induced tumor cell expression of MHC class I, IFNAR1 and PD-L1 molecules and secretion of CXCL10 in vitro [file 41422_2019_224_MOESM3_ESM.pdf]

Figure S3

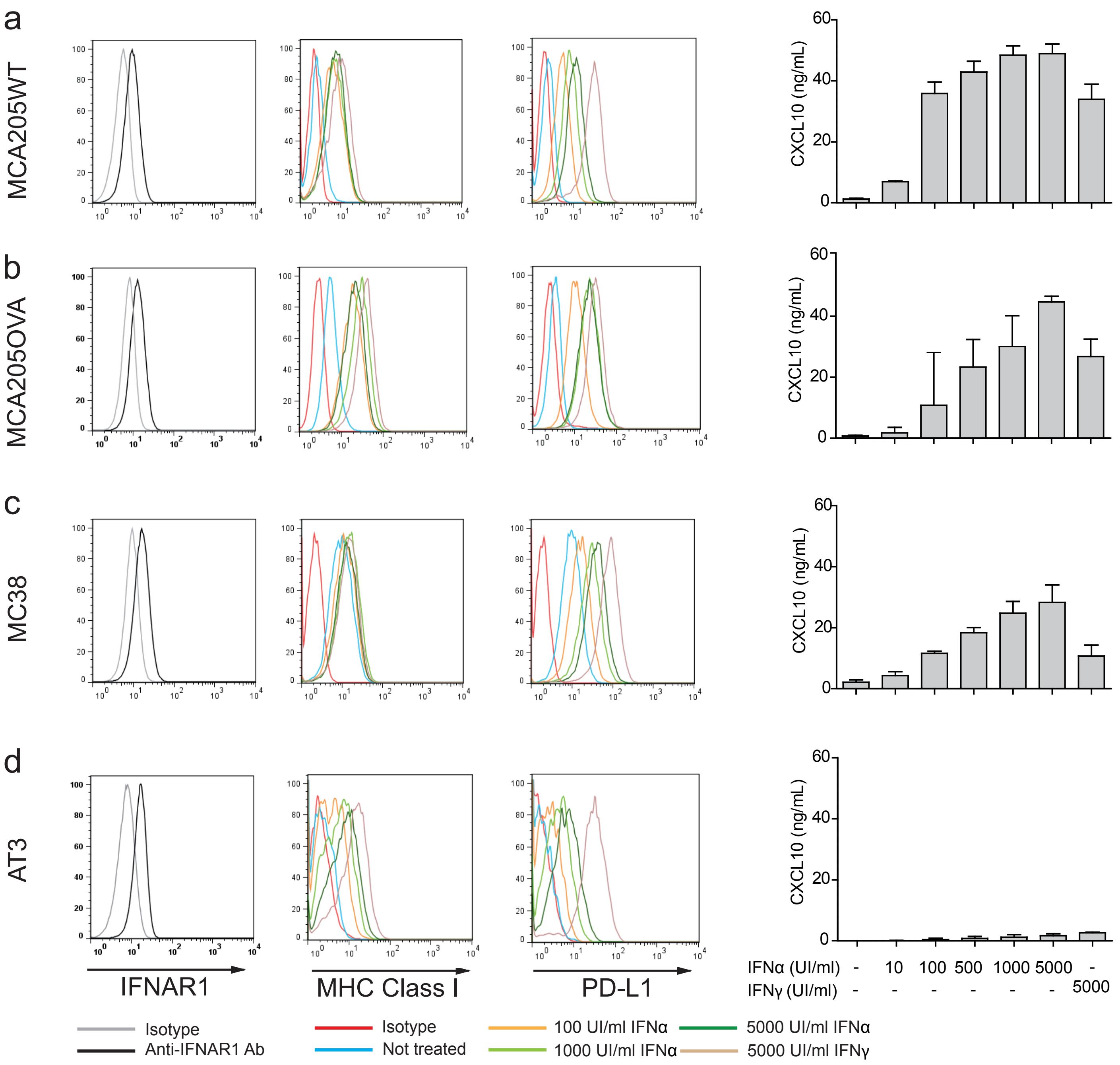
a
b
c
d
MCA205WT
MCA205OVA
MC38
AT3
CXCL10 (ng/mL)
IFNα (UI/ml) - 10 100 500 1000 5000 -
IFNγ (UI/ml) - - - - - - 5000
IFNAR1
MHC Class I
PD-L1
Isotype
Anti-IFNAR1 Ab
Isotype
Not treated
100 UI/ml IFNα
1000 UI/ml IFNα
5000 UI/ml IFNα
5000 UI/ml IFNγ

Supplementary information, Fig S3. IFN-induced tumor cell expression of MHC class I, IFNAR1 and PD-L1 molecules and secretion of CXCL10 *in vitro*.

MCA205WT (a), MCA205OVA (b), MC38 (c) and AT3 (d) cell lines were assessed for IFNAR1 (left panels), MHC Class I (middle) and PD-L1 (right) expression levels by flow cytometry using specific antibodies and appropriate isotype controls after gating on live cells, at baseline and/or after 24 hrs stimulation with increasing concentrations of IFNα. One single concentration of IFNγ has been used as a positive control. Supernatant was collected to measure the concentration of CXCL10 by ELISA. For overlays, a typical experiment is depicted out of 3-4 yielding similar results including biological replicates for each experiment. CXCL10 concentration represents a pool of 3-4 experiments including biological replicates for each experiment. Means ± SEM are shown.
