# Supplementary material for: Sustained Type I interferon signaling as a mechanism of resistance to PD-1 blockade
Source: Cell Res. 2019 Sep 3;29(10):846–61. doi: 10.1038/s41422-019-0224-x (PMC6796942; doi:10.1038/s41422-019-0224-x)
Supplement: Supplementary file 4 — Supplementary information, Fig S4. IFNβ and ISG gene expression levels in CD45- cells isolated from MCA205WT and MC38 tumors [file 41422_2019_224_MOESM4_ESM.pdf]

Figure S4

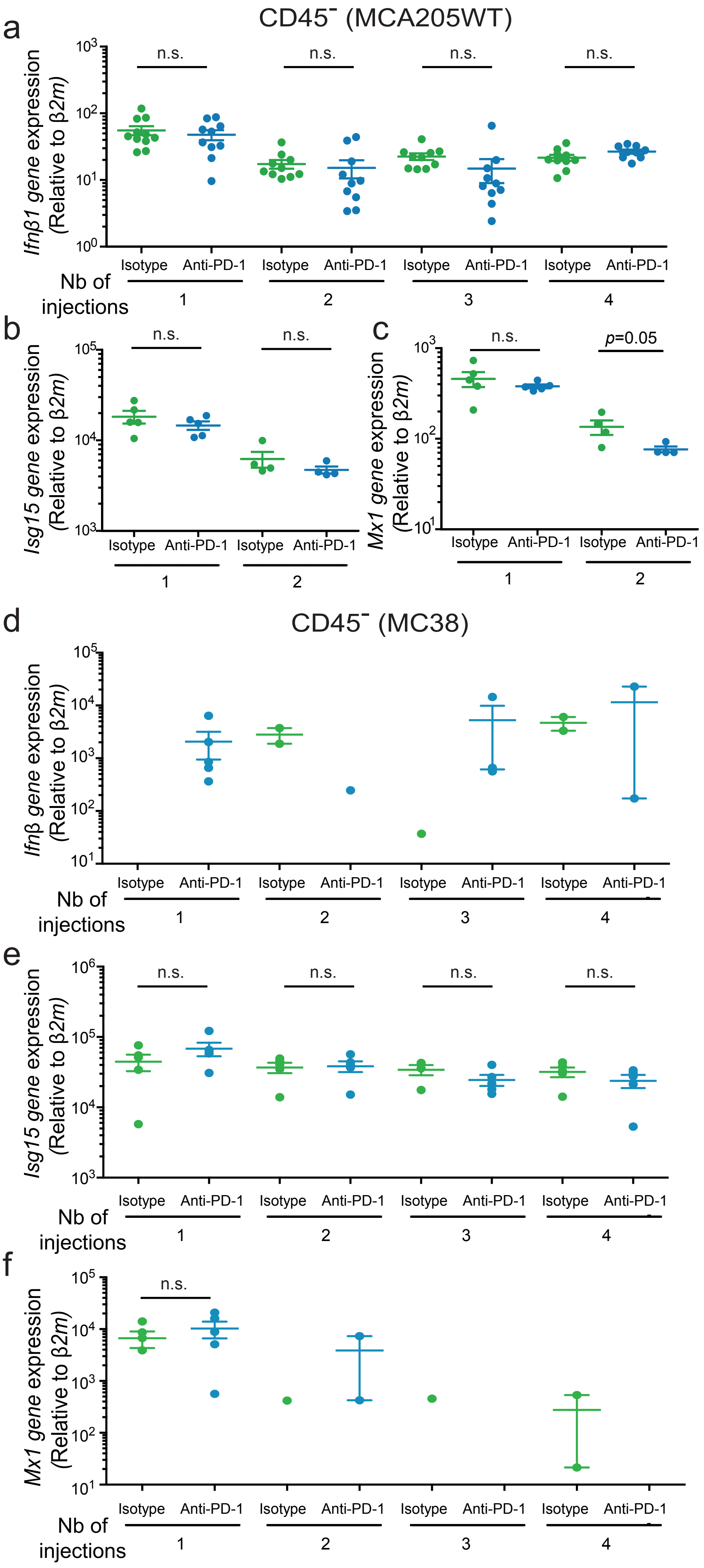

a
CD45⁻ (MCA205WT)
Ifnβ1 gene expression (Relative to β2m)
10³
10²
10¹
10⁰
n.s.
n.s.
n.s.
n.s.
Isotype Anti-PD-1 Isotype Anti-PD-1 Isotype Anti-PD-1 Isotype Anti-PD-1
Nb of injections
1 2 3 4
b
Isg15 gene expression (Relative to β2m)
10⁵
10⁴
10³
n.s.
n.s.
Isotype Anti-PD-1 Isotype Anti-PD-1
1 2
c
Mx1 gene expression (Relative to β2m)
10³
10²
10¹
n.s.
p=0.05
Isotype Anti-PD-1 Isotype Anti-PD-1
1 2
d
CD45⁻ (MC38)
Ifnβ gene expression (Relative to β2m)
10⁵
10⁴
10³
10²
10¹
Isotype Anti-PD-1 Isotype Anti-PD-1 Isotype Anti-PD-1 Isotype Anti-PD-1
Nb of injections
1 2 3 4
e
Isg15 gene expression (Relative to β2m)
10⁶
10⁵
10⁴
10³
n.s.
n.s.
n.s.
n.s.
Isotype Anti-PD-1 Isotype Anti-PD-1 Isotype Anti-PD-1 Isotype Anti-PD-1
Nb of injections
1 2 3 4
f
Mx1 gene expression (Relative to β2m)
10⁵
10⁴
10³
10²
10¹
n.s.
Isotype Anti-PD-1 Isotype Anti-PD-1 Isotype Anti-PD-1 Isotype Anti-PD-1
Nb of injections
1 2 3 4

Supplementary information, Fig S4. IFNβ and ISG gene expression levels in CD45^{-} cells isolated from MCA205WT and MC38 tumors. (a-f) *In vivo* experiments. Flow cytometry sorting of CD45^{-} live fractions from the TME of MCA205WT (a-c) or MC38 (d-f) tumors 48 hrs after 1, 2, 3 or 4 i.p. administrations of anti-PD-1 or isotype control mAbs. Relative expression of *Ifnβ1* (a and d) and IFN-sensitive gene products (b-c and e-f) quantified by qRT-PCR. Statistical analyses were performed using unpaired t-tests to compare transcription levels between the anti-PD-1 and isotype control treated groups for each time point. Each dot represents 1 mouse with 5 mice per time point and per experiment. Graphs represent 1 representative experiment out of 2-3 independent experiments (MC38, time points 1 and 2, b-c), 1 experiment (MC38, time points 3 and 4) or are the pool of 2-3 independent experiments (a). *Ifnβ* and *Mx1* gene expression levels were not detected in many samples (d and f). n.s.: not significant. Means ± SEM are represented.
