# Supplementary material for: Sustained Type I interferon signaling as a mechanism of resistance to PD-1 blockade
Source: Cell Res. 2019 Sep 3;29(10):846–61. doi: 10.1038/s41422-019-0224-x (PMC6796942; doi:10.1038/s41422-019-0224-x)
Supplement: Supplementary file 5 — Supplementary information, Fig S5. Global PD-L1 expression upregulation during PD-1 blockade and post IFN stimulation [file 41422_2019_224_MOESM5_ESM.pdf]

Figure S5

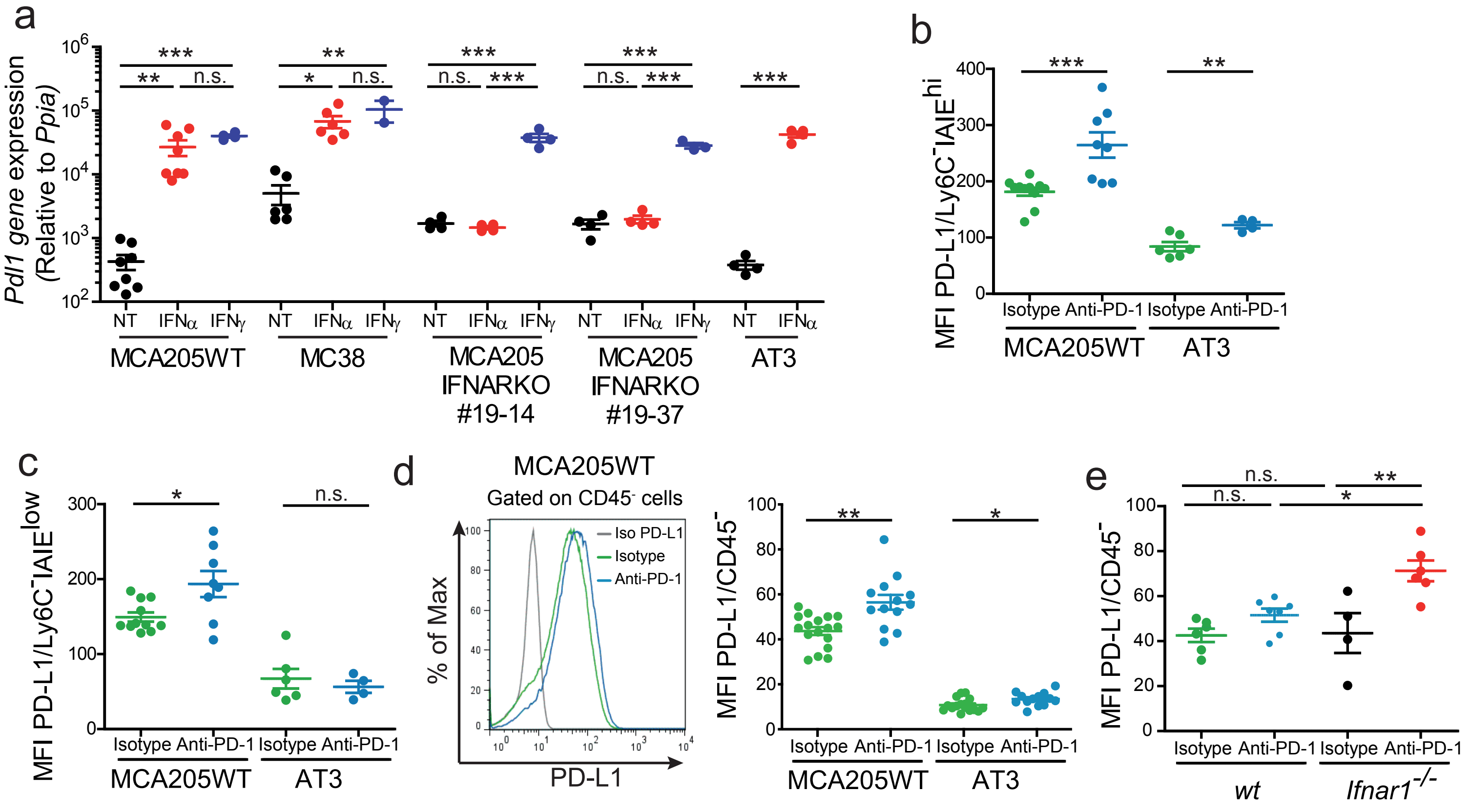

a
Pdl1 gene expression (Relative to Ppia)
NT IFNα IFNγ
MCA205WT
MC38
MCA205 IFNARKO #19-14
MCA205 IFNARKO #19-37
AT3
b
MFI PD-L1/Ly6C-IAIEhi
Isotype Anti-PD-1 Isotype Anti-PD-1
MCA205WT AT3
c
MFI PD-L1/Ly6C-IAIElow
Isotype Anti-PD-1 Isotype Anti-PD-1
MCA205WT AT3
d
MCA205WT
Gated on CD45- cells
Iso PD-L1
Isotype
Anti-PD-1
% of Max
PD-L1
MFI PD-L1/CD45-
Isotype Anti-PD-1 Isotype Anti-PD-1
MCA205WT AT3
e
MFI PD-L1/CD45-
Isotype Anti-PD-1 Isotype Anti-PD-1
wt Ifnar1-/-

Supplementary information, Fig S5. Global PD-L1 expression upregulation during PD-1 blockade and post IFN stimulation.

(a) Relative expression of *Pdl1* quantified by qRT-PCR following stimulations of various tumor cell lines with either IFNα or IFNγ. Each dot represents 1 stimulated sample and graphs represent 1 experiment or are the pool of 2-3 independent experiments including biological replicates for each experiment. Unpaired t-tests were used to compare two groups or ANOVA statistical tests and pairwise comparisons with Bonferroni adjustment were adopted for more than two groups. (b-d) Mean Fluorescent Intensity (MFI) of PD-L1 expression at the surface of myeloid cells (gated on live $CD45^+CD11b^+$) and $CD45^-$ live cells assessed by flow cytometry 48 hrs after the last injection of anti-PD-1 or its isotype control mAbs in MCA205WT and AT3 tumor models. Representative histogram of PD-L1 expression on $CD45^-$ cells isolated from MCA205WT tumors is shown (d). Statistical analyses were performed using unpaired t-tests. (e) MFI of PD-L1 on $CD45^-$ cells from MCA205WT tumors inoculated in WT or *Ifnar1*$^{-/-}$ mice. Statistical analyses were performed using ANOVA statistical test and pairwise comparisons with Bonferroni adjustment. Graphs (b-e) represent 1 or are the pool of 2-4 independent experiments with 5-6 mice per group and per experiment. Each dot corresponds to one mouse. *p<0.05, **p<0.01, ***p<0.001, n.s.: not significant. Means $\pm$ SEM are represented.
