# Supplementary material for: Sustained Type I interferon signaling as a mechanism of resistance to PD-1 blockade
Source: Cell Res. 2019 Sep 3;29(10):846–61. doi: 10.1038/s41422-019-0224-x (PMC6796942; doi:10.1038/s41422-019-0224-x)
Supplement: Supplementary file 6 — Supplementary information, Fig S6. CD80/86 engagement or tumoral PD-L1 expression are not associated with resistance to anti-PD-1 therapy [file 41422_2019_224_MOESM6_ESM.pdf]

Figure S6

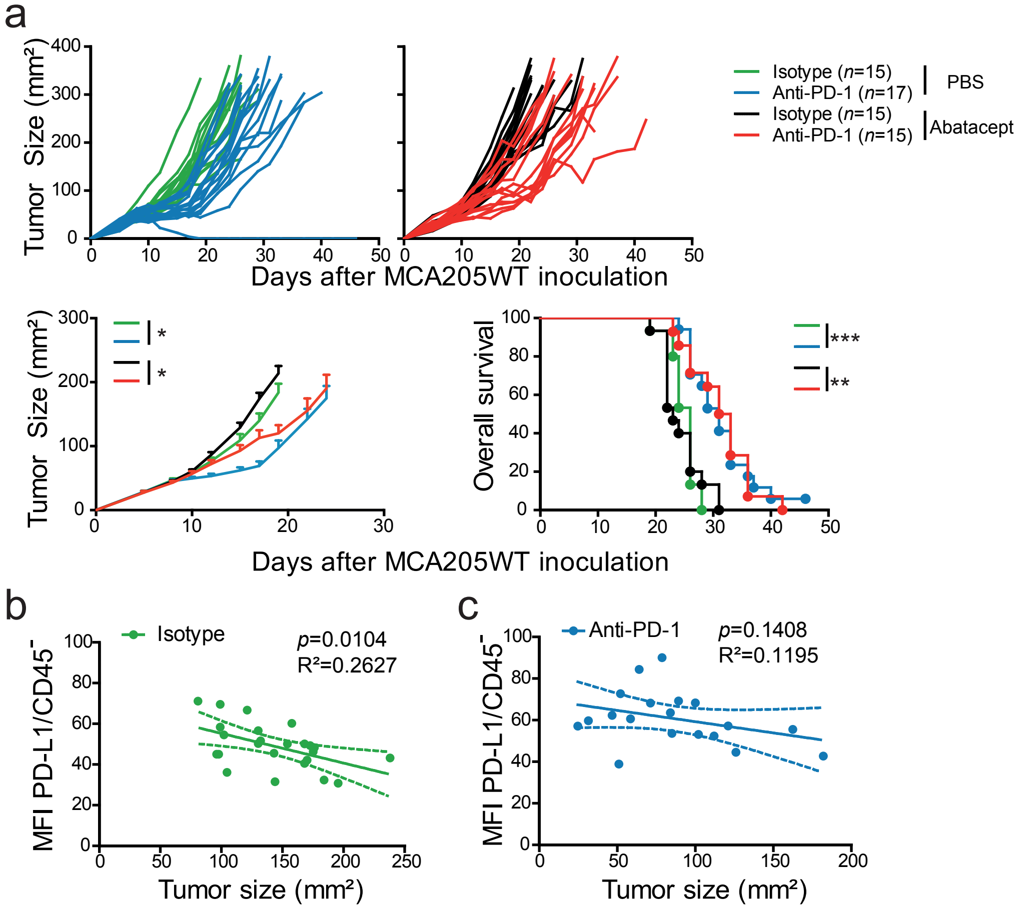
a
Tumor Size (mm²)
Days after MCA205WT inoculation
Isotype (n=15)
Anti-PD-1 (n=17)
PBS
Isotype (n=15)
Anti-PD-1 (n=15)
Abatacept
*
*
Overall survival
***
**
b
Isotype
p=0.0104
R²=0.2627
MFI PD-L1/CD45⁻
Tumor size (mm²)
c
Anti-PD-1
p=0.1408
R²=0.1195
MFI PD-L1/CD45⁻
Tumor size (mm²)

Supplementary information, Fig S6. CD80/86 engagement or tumoral PD-L1 expression are not associated with resistance to anti-PD-1 therapy.

(a) Tumor growth kinetics and Kaplan Meier survival curves of MCA205WT tumor bearing mice treated concomitantly with or without Abatacept and anti-PD-1 (and their respective controls). Statistical analyses were performed using the specific software detailed in the Materials and Methods. (b-c) Correlations between the MFI of PD-L1 monitored on the surface of $CD45^-$ cells isolated from MCA205WT tumors collected from isotype (b) and anti-PD-1 treated mice (c) and their respective tumor size at sacrifice. Pearson correlation was used to determine the correlation between the two variables. Graphs depict results of 2 (a) or 3 (b-c) independent experiments encompassing 5-11 mice/group and per experiment; each line or dot corresponds to one mouse. *$p<0.05$, **$p<0.01$, ***$p<0.001$. Means ± SEM are represented.
