# Supplementary material for: Sustained Type I interferon signaling as a mechanism of resistance to PD-1 blockade
Source: Cell Res. 2019 Sep 3;29(10):846–61. doi: 10.1038/s41422-019-0224-x (PMC6796942; doi:10.1038/s41422-019-0224-x)
Supplement: Supplementary file 7 — Supplementary information, Fig S7. Flow cytometric analyses of ARG1 and NOS2 in BMDCs and BMMCs [file 41422_2019_224_MOESM7_ESM.pdf]

Figure S7

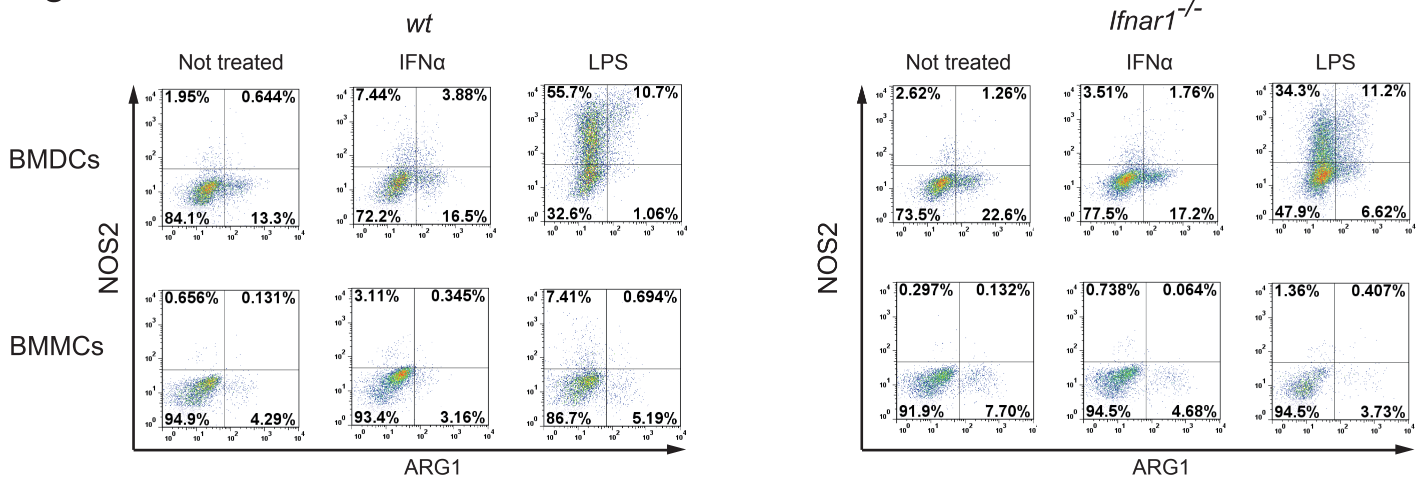
wt
Ifnar1-/-
Not treated
IFNα
LPS
BMDCs
BMMCs
NOS2
ARG1
1.95%
0.644%
84.1%
13.3%
7.44%
3.88%
72.2%
16.5%
55.7%
10.7%
32.6%
1.06%
0.656%
0.131%
94.9%
4.29%
3.11%
0.345%
93.4%
3.16%
7.41%
0.694%
86.7%
5.19%
2.62%
1.26%
73.5%
22.6%
3.51%
1.76%
77.5%
17.2%
34.3%
11.2%
47.9%
6.62%
0.297%
0.132%
91.9%
7.70%
0.738%
0.064%
94.5%
4.68%
1.36%
0.407%
94.5%
3.73%

Supplementary information, Fig S7. Flow cytometric analyses of ARG1 and NOS2 in BMDCs and BMMCs.

Representative flow cytometry analysis of intracellular staining with anti-Arginase 1 and anti-NOS2 Abs in BMDCs or BMMCs from WT and *Ifnar1*$^{-/-}$ mice stimulated *in vitro* with LPS or IFNα for 24 hrs. Biological replicates were examined for each condition.
