# Supplementary material for: Sustained Type I interferon signaling as a mechanism of resistance to PD-1 blockade
Source: Cell Res. 2019 Sep 3;29(10):846–61. doi: 10.1038/s41422-019-0224-x (PMC6796942; doi:10.1038/s41422-019-0224-x)
Supplement: Supplementary file 8 — Supplementary information, Fig S8. Expressions of Nos1 and Nos3 are not modulated in the TME during the course of anti-PD-1 mAbs [file 41422_2019_224_MOESM8_ESM.pdf]

Figure S8

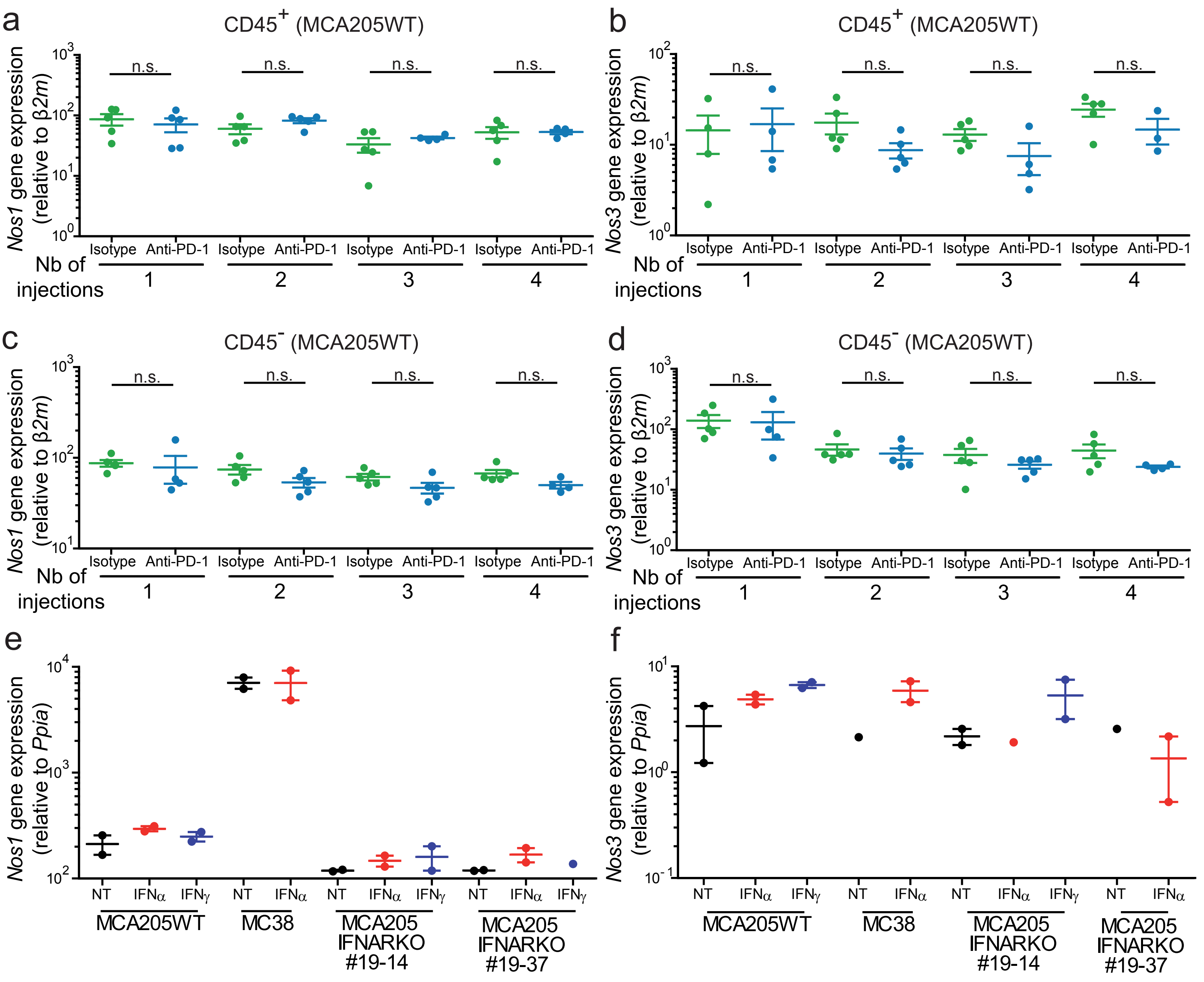
a
CD45+ (MCA205WT)
Nos1 gene expression (relative to β2m)
n.s.
Isotype
Anti-PD-1
Nb of injections
1
2
3
4
b
CD45+ (MCA205WT)
Nos3 gene expression (relative to β2m)
c
CD45- (MCA205WT)
Nos1 gene expression (relative to β2m)
d
CD45- (MCA205WT)
Nos3 gene expression (relative to β2m)
e
Nos1 gene expression (relative to Ppia)
NT
IFNα
IFNγ
MCA205WT
MC38
MCA205 IFNARKO #19-14
MCA205 IFNARKO #19-37
f
Nos3 gene expression (relative to Ppia)

Supplementary information, Fig S8. Expressions of *Nos1* and *Nos3* are not modulated in the TME during the course of anti-PD-1 mAbs.

(a-d) *In vivo* experiments. $CD45^+$ and $CD45^-$ fractions from MCA205WT tumors were cell sorted 48 hrs after anti-PD-1 mAb (or isotype control) injections. qRT-PCR analyses of *Nos1* (a and c) and *Nos3* (b and d) in $CD45^+$ (a and b) and $CD45^-$ (c and d) fractions. (e-f) *In vitro* assays. *Nos1* (e) and *Nos3* (f) expression levels were determined in various tumor cell lines post IFNα or IFNγ stimulation. Graphs depict 1 experiment with biological replicates. Each dot represents 1 tumor or 1 stimulation condition. Statistical analyses were performed using unpaired t-tests to compare two groups or ANOVA statistical tests and pairwise comparisons with Bonferroni adjustment for more than two groups. n.s.: not significant. Means $\pm$ SEM are represented.
