# Supplementary material for: Sustained Type I interferon signaling as a mechanism of resistance to PD-1 blockade
Source: Cell Res. 2019 Sep 3;29(10):846–61. doi: 10.1038/s41422-019-0224-x (PMC6796942; doi:10.1038/s41422-019-0224-x)
Supplement: Supplementary file 9 — Supplementary information, Fig S9. Therapeutic resistance to anti-PD-1 mAb is Arginase 1 independent [file 41422_2019_224_MOESM9_ESM.pdf]

Figure S9

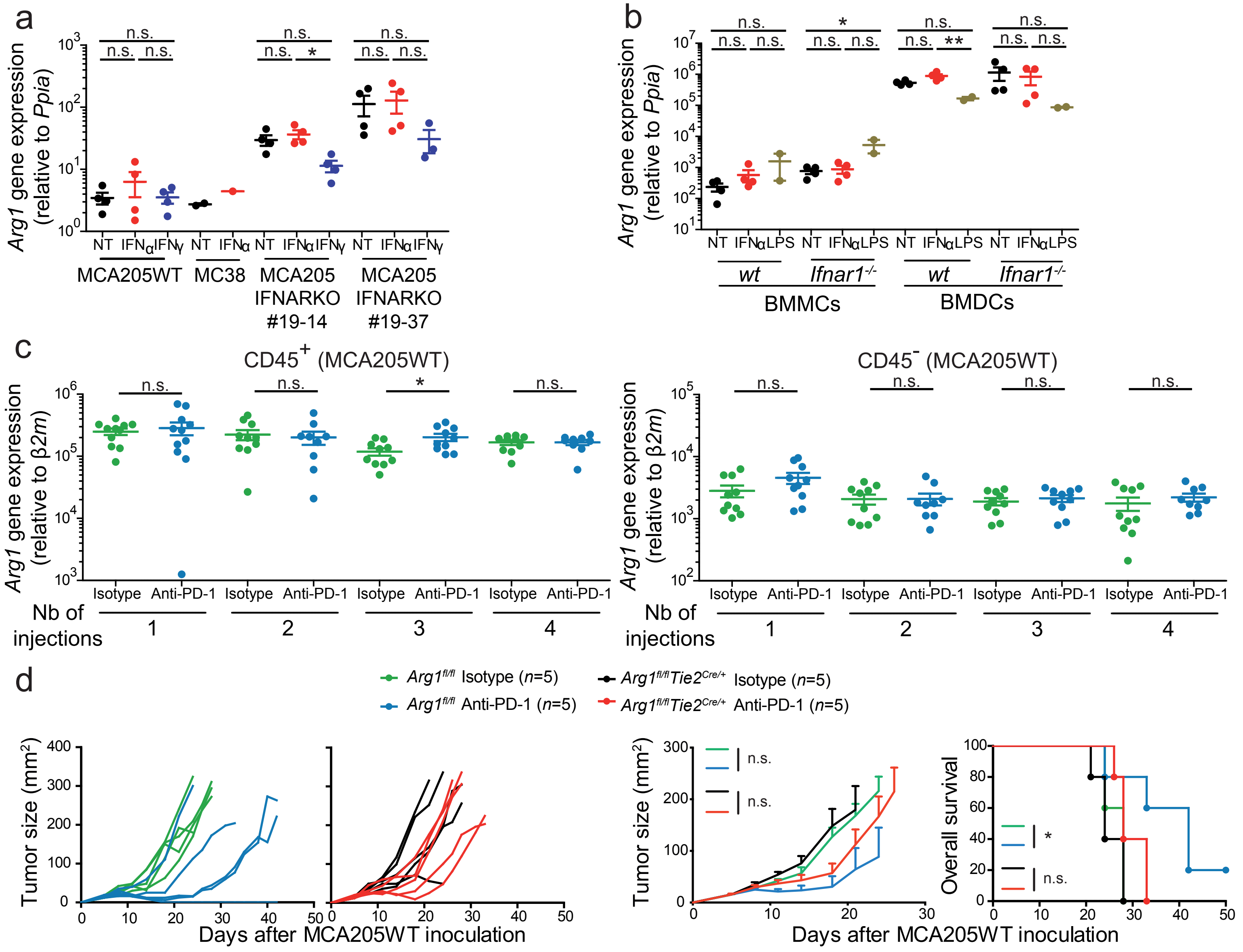
a
Arg1 gene expression (relative to Ppia)
n.s.
*
NT IFNα IFNγ NT IFNα NT IFNα IFNγ NT IFNα IFNγ
MCA205WT MC38 MCA205 IFNARKO #19-14 MCA205 IFNARKO #19-37
b
**
NT IFNα LPS NT IFNα LPS NT IFNα LPS NT IFNα LPS
wt Ifnar1-/- wt Ifnar1-/-
BMMCs BMDCs
c
CD45+ (MCA205WT)
CD45- (MCA205WT)
Arg1 gene expression (relative to β2m)
Isotype Anti-PD-1
Nb of injections
1 2 3 4
d
Arg1fl/fl Isotype (n=5)
Arg1fl/fl Anti-PD-1 (n=5)
Arg1fl/flTie2Cre/+ Isotype (n=5)
Arg1fl/flTie2Cre/+ Anti-PD-1 (n=5)
Tumor size (mm2)
Days after MCA205WT inoculation
Overall survival

Supplementary information, Fig S9. Therapeutic resistance to anti-PD-1 mAb is Arginase 1 independent.

(a-b) *In vitro* assays. Relative expression of *Arg1* quantified by qRT-PCR following stimulations of various tumor cell lines or BMDCs and BMMCs with either IFNα, IFNγ or LPS. Each dot represents one stimulated sample and graphs represent 1 experiment or are the pool of 2 independent experiments including biological replicates for each experiment. Statistical analyses were performed using unpaired t-tests to compare two groups or ANOVA statistical tests and pairwise comparisons with Bonferroni adjustment for more than two groups. (c) *In vivo* experiments. Cell sorting by flow cytometry of $CD45^+$ (left panel) and $CD45^-$ (right panel) fractions from the TME of MCA205WT 48 hrs after 1, 2, 3 or 4 i.p. administrations of anti-PD-1 (or isotype control) mAbs followed by qRT-PCR quantifying the *Arg1* relative expression. Each dot represents 1 tumor. Graphs represent the pool of 2 experiments with 4-5 mice per group, per time point and per experiment. (d) Tumor growth kinetics and Kaplan Meier survival curves of $Arg1^{fl/fl}$ and $Arg1^{fl/fl}Tie2^{Cre/+}$ mice inoculated with the MCA205WT tumor model and treated with anti-PD-1 or its isotype control mAbs. Statistical analyses were performed using the specific software detailed in the Materials and Methods. Data are representative of 1 experiment with 5 mice per group. *$p<0.05$, **$p<0.01$, n.s.: not significant.
