# Supplementary material for: Sustained Type I interferon signaling as a mechanism of resistance to PD-1 blockade
Source: Cell Res. 2019 Sep 3;29(10):846–61. doi: 10.1038/s41422-019-0224-x (PMC6796942; doi:10.1038/s41422-019-0224-x)
Supplement: Supplementary file 10 — Supplementary information, Fig S10. NOS2 upregulation post anti-PD-1 therapy in melanoma patients [file 41422_2019_224_MOESM10_ESM.pdf]

Figure S10

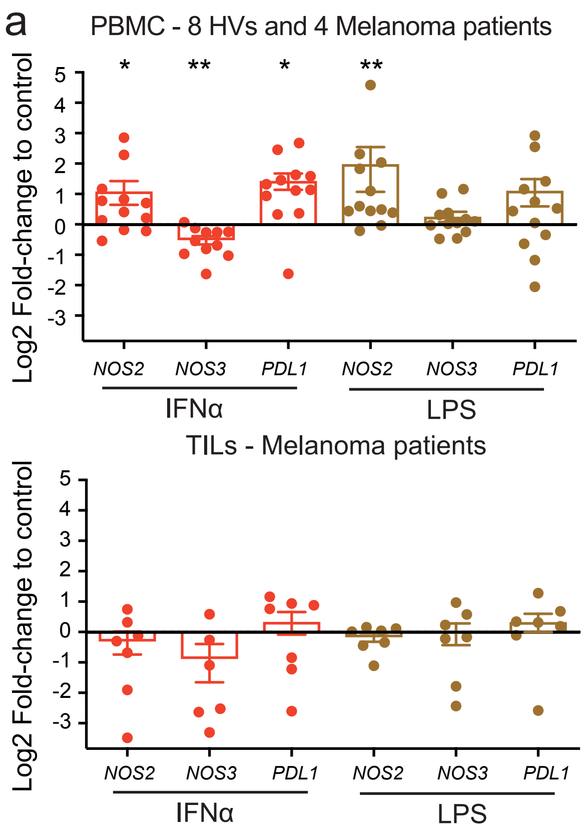
a
PBMC - 8 HVs and 4 Melanoma patients
Log2 Fold-change to control
* ** * **
NOS2 NOS3 PDL1 NOS2 NOS3 PDL1
IFNα
LPS
TILs - Melanoma patients
Log2 Fold-change to control
NOS2 NOS3 PDL1 NOS2 NOS3 PDL1
IFNα
LPS

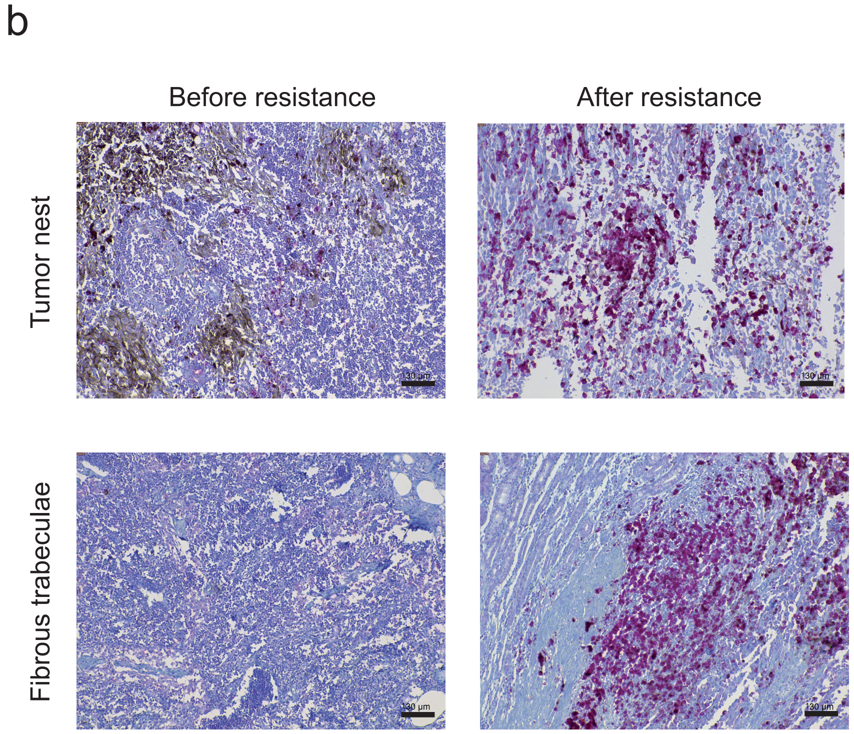
b
Before resistance
After resistance
Tumor nest
Fibrous trabeculae

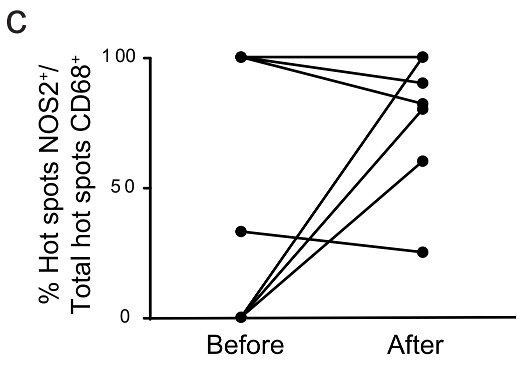
c
% Hot spots NOS2+/ Total hot spots CD68+
100
50
0
Before
After

Supplementary information, Fig S10. NOS2 upregulation post anti-PD-1 therapy in melanoma patients.

(a) Relative expression of *NOS*2, *NOS3* and *PDL1* quantified by qRT-PCR following stimulations by type I IFN and LPS in peripheral blood mononuclear cells (PBMC) from 8 healthy volunteers and 4 melanoma patients (upper panel) together with TILs from 7 stage III/IV melanoma patients (lower panel). Log_2 fold-change relative to untreated control is represented. Each dot represents one healthy volunteer or patient. Means ± SEM are shown. Statistical analyses were performed using Wilcoxon matched-pairs signed rank test (compared to untreated control conditions). **p*<0.05, ***p*<0.01. (b) Representative IHC images from tumor samples from a melanoma patient are shown for intratumor and peritumor areas, before and after anti-PD-1 therapy. NOS2-positive cells are stained red. Magnification = 100X. (c) Quantification of NOS2-positive hotspots relative to the total number of CD68 positive hot spots in each sample. Each dot represents 1 patient (n=7). Results are presented as percentages.
