# Supplementary material for: Sustained Type I interferon signaling as a mechanism of resistance to PD-1 blockade
Source: Cell Res. 2019 Sep 3;29(10):846–61. doi: 10.1038/s41422-019-0224-x (PMC6796942; doi:10.1038/s41422-019-0224-x)
Supplement: Supplementary file 11 — Supplementary information, Fig S11 [file 41422_2019_224_MOESM11_ESM.pdf]

Figure S11

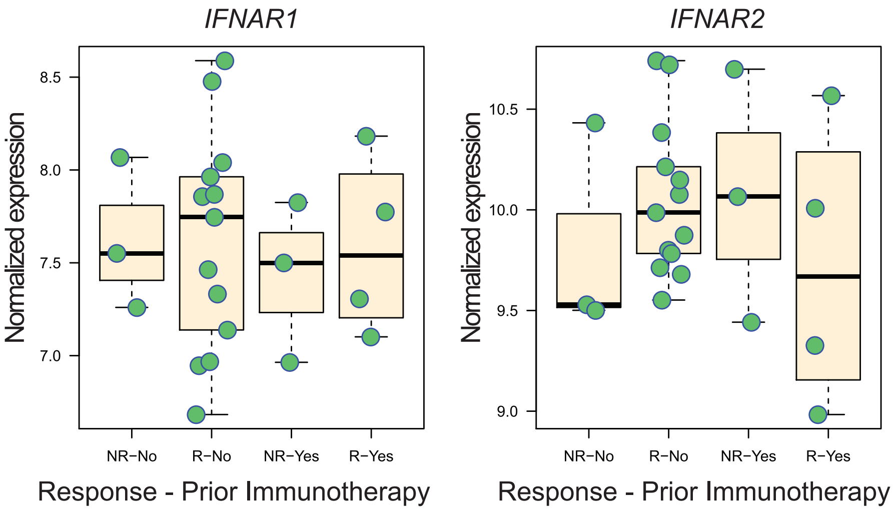

IFNAR1
IFNAR2
Normalized expression
8.5
8.0
7.5
7.0
10.5
10.0
9.5
9.0
NR−No
R−No
NR−Yes
R−Yes
Response - Prior Immunotherapy

Supplementary information, Fig S11.

Boxplots of *IFNAR1* (left) and *IFNAR2* (right) gene expression in pre-treatment samples stratified by response to subsequent combination immune checkpoint blockade (R=responder, NR=non-responder) and receipt of prior immunotherapy (Yes/No) showing no substantial differences between any subgroup.
