# Supplementary material for: Sustained Type I interferon signaling as a mechanism of resistance to PD-1 blockade
Source: Cell Res. 2019 Sep 3;29(10):846–61. doi: 10.1038/s41422-019-0224-x (PMC6796942; doi:10.1038/s41422-019-0224-x)
Supplement: Supplementary file 12 — Table S1 : Genes list found differentially expressed post anti-PD-1 treatment in CD45+ cells [file 41422_2019_224_MOESM12_ESM.pdf]

Table S1 : Genes list found differentially expressed post anti-PD-1 treatment in $CD45^{+}$ cells

Downregulated genes		
GeneName	logFC	p.value
Igh-VJ558	-0,588147346	0,000770031
Tns1	-0,630399079	0,001605814
Bcar3	-0,608438415	0,001947286
Olfr111	-1,005908493	0,002208479
Mgl2	-1,130733319	0,003047944
Tcrg-V1	-0,768636655	0,003107205
COX1	-0,606473561	0,003143469
Zdhhc14	-0,650586235	0,005076849
Zranb1	-0,616840727	0,005622662
Ccl17	-1,268308284	0,006575942
Plxdc2	-0,742127905	0,00738555
Gm8680	-0,651866875	0,010920686
Retnlg	-0,850612057	0,011612273
Plet1	-0,941079444	0,013157657
Hp1bp3	-0,592604523	0,014476284
Il1a	-0,875921079	0,019232187
Zkscan3	-1,005924357	0,019328747
Clec4n	-0,59232767	0,021008517
Igkv8-30	-0,720322357	0,023857958
Anapc1	-0,861952817	0,029778845
Mmp12	-0,804784063	0,033921738
Tcrg-V4	-0,787778644	0,03608202

**Upregulated genes**		
GeneName	logFC	p.value
C4b	0,621016055	1,46378E-05
Slc4a11	1,046422288	0,000123917
Hk3	0,688772445	0,000192591
Slamf8	0,648146459	0,000361821
Nos2	1,219194586	0,000803245
Irg1	0,795357716	0,001032831
Smpdl3b	0,669662873	0,001313525
Slco2a1	0,767029039	0,002081427
Gbp2b	1,536388287	0,002544813
Abhd16a	0,754939361	0,004127977
Pdpk1	0,597633371	0,006370427
Iigp1	0,621115521	0,006885211
Riok3	0,68263627	0,010174209
Rnf220	1,281218931	0,012430493
Ly6i	0,602969515	0,012596897
Xpo7	0,640173124	0,01621813
Gbf1	0,85268858	0,022081152
Dhrs1	0,707689893	0,022172429
Huwe1	0,865743918	0,02643558
