# Supplementary material for: Sustained Type I interferon signaling as a mechanism of resistance to PD-1 blockade
Source: Cell Res. 2019 Sep 3;29(10):846–61. doi: 10.1038/s41422-019-0224-x (PMC6796942; doi:10.1038/s41422-019-0224-x)
Supplement: Supplementary file 13 — Table S2. Related to Figure S8 (B-C). Patient ’s characteristics [file 41422_2019_224_MOESM13_ESM.pdf]

Table S2. Related to Figure S8 (B-C). Patient's characteristics

		All - N (%)	Germany - N (%)	Italy - N (%)
**Gender**	F	0 (0)	0 (0)	0 (0)
	M	7 (100)	3 (100)	4 (100)
**Age at the time of the treatment (years, Median [Min;Max])**		57 [38;75]	74 [48;75]	52 [38;58]
**Tumor stage**	Ⅲ	2 (29)	2 (66)	0 (0)
	Ⅳ	5 (71)	1 (33)	4 (100)
**Previous treatments**	No	2 (29)	2 (66)	0 (0)
	Yes*	5 (71)	1 (33)	4 (100)
**Tumor response**	PR	3 (43)	0 (0)	3 (75)
	SD	1 (14)	1 (33)	0 (0)
	PD	3 (43)	2 (66)	1 (25)
**Toxicity**	No	6 (86)	2 (67)	4 (100)
	Yes	1 (14)	1 (33)	0 (0)

* All previous treatments were composed of at least one line of Immune checkpoint blocker
PR: partial response. SD: stable disease. PD: progressive disease
