# Supplementary material for: Sustained Type I interferon signaling as a mechanism of resistance to PD-1 blockade
Source: Cell Res. 2019 Sep 3;29(10):846–61. doi: 10.1038/s41422-019-0224-x (PMC6796942; doi:10.1038/s41422-019-0224-x)
Supplement: Supplementary file 14 — Table S3: Details of the custom-designed 795-gene codeset [file 41422_2019_224_MOESM14_ESM.pdf]

Table S3: Details of the custom-designed 795-gene codeset

CodeClass	Name	Accession
Positive	POS_A	ERCC_00117.1
Positive	POS_B	ERCC_00112.1
Positive	POS_C	ERCC_00002.1
Positive	POS_D	ERCC_00092.1
Positive	POS_E	ERCC_00035.1
Positive	POS_F	ERCC_00034.1
Negative	NEG_A	ERCC_00096.1
Negative	NEG_B	ERCC_00041.1
Negative	NEG_C	ERCC_00019.1
Negative	NEG_D	ERCC_00076.1
Negative	NEG_E	ERCC_00098.1
Negative	NEG_F	ERCC_00126.1
Negative	NEG_G	ERCC_00144.1
Negative	NEG_H	ERCC_00154.1
Endogenous	ABCF1	NM_001025091.1
Endogenous	ACKR2	NM_001296.3
Endogenous	ACKR4	NM_016557.2
Endogenous	ACO2	NM_001098.2
Endogenous	ACTB	NM_001101.2
Endogenous	ACVR1C	NM_145259.2
Endogenous	ACVR2A	NM_001616.3
Endogenous	ACVR2B	NM_001106.2
Endogenous	ADA	NM_000022.2
Endogenous	AIM2	NM_004833.1
Endogenous	ALAS1	NM_000688.4
Endogenous	ALCAM	NM_001627.3
Endogenous	AMICA1	NM_153206.2
Endogenous	AMIGO1	NM_020703.2
Endogenous	AMIGO2	NM_001143668.1
Endogenous	AMIGO3	NM_198722.2
Endogenous	ANGPT1	NM_001146.3
Endogenous	ANGPT2	NM_001118887.1
Endogenous	ANGPT4	NM_015985.2
Endogenous	AOC3	NM_003734.2
Endogenous	ARG1	NM_000045.2
Endogenous	ARG2	NM_001172.3
Endogenous	BATF3	NM_018664.2
Endogenous	BCAM	NM_005581.3
Endogenous	BCAN	NM_198427.1
Endogenous	BECN1	NM_003766.2
Endogenous	BGN	NM_001711.3
Endogenous	BID	NM_197966.1
Endogenous	BMPR1A	NM_004329.2
Endogenous	BMPR1B	NM_001203.1

Endogenous	BMPR2	NM_001204.5
Endogenous	BST2	NM_004335.2
Endogenous	C3	NM_000064.2
Endogenous	C5	NM_001735.2
Endogenous	CADM1	NM_014333.3
Endogenous	CADM2	NM_001167674.1
Endogenous	CADM3	NM_001127173.1
Endogenous	CADM4	NM_145296.1
Endogenous	CALR	NM_004343.2
Endogenous	CANX	NM_001024649.1
Endogenous	CARD11	NM_032415.2
Endogenous	CASP1	NM_033294.2
Endogenous	CASP3	NM_004346.3
Endogenous	CASP4	NM_001225.3
Endogenous	CCL1	NM_002981.1
Endogenous	CCL11	NM_002986.2
Endogenous	CCL13	NM_005408.2
Endogenous	CCL14	NM_032963.3
Endogenous	CCL15	NM_032965.4
Endogenous	CCL16	NM_004590.2
Endogenous	CCL17	NM_002987.2
Endogenous	CCL18	NM_002988.2
Endogenous	CCL19	NM_006274.2
Endogenous	CCL2	NM_002982.3
Endogenous	CCL20	NM_004591.2
Endogenous	CCL21	NM_002989.2
Endogenous	CCL22	NM_002990.3
Endogenous	CCL23	NM_145898.1
Endogenous	CCL24	NM_002991.2
Endogenous	CCL25	NM_005624.2
Endogenous	CCL26	NM_006072.4
Endogenous	CCL27	NM_006664.2
Endogenous	CCL28	NM_148672.2
Endogenous	CCL3	NM_002983.2
Endogenous	CCL4	NM_002984.2
Endogenous	CCL5	NM_002985.2
Endogenous	CCL7	NM_006273.2
Endogenous	CCL8	NM_005623.2
Endogenous	CCR1	NM_001295.2
Endogenous	CCR10	NM_016602.1
Endogenous	CCR2	NM_000647.3
Endogenous	CCR3	NM_001837.2
Endogenous	CCR4	NM_005508.4
Endogenous	CCR5	NM_000579.1
Endogenous	CCR6	NM_004367.5
Endogenous	CCR7	NM_001838.2

Endogenous	CCR8	NM_005201.2
Endogenous	CD109	NM_133493.3
Endogenous	CD14	NM_000591.2
Endogenous	CD19	NM_001770.4
Endogenous	CD1A	NM_001763.2
Endogenous	CD1B	NM_001764.2
Endogenous	CD1C	NM_001765.2
Endogenous	CD1D	NM_001766.3
Endogenous	CD1E	NM_001042583.1
Endogenous	CD200	NM_005944.5
Endogenous	CD200R1	NM_138939.2
Endogenous	CD209	NM_021155.3
Endogenous	CD22	NM_001771.2
Endogenous	CD244	NM_016382.2
Endogenous	CD247	NM_198053.1
Endogenous	CD274	NM_014143.2
Endogenous	CD28	NM_001243078.1
Endogenous	CD34	NM_001773.2
Endogenous	CD3D	NM_000732.4
Endogenous	CD3E	NM_000733.2
Endogenous	CD3G	NM_000073.2
Endogenous	CD4	NM_000616.4
Endogenous	CD40	NM_152854.2
Endogenous	CD40LG	NM_000074.2
Endogenous	CD44	NM_001001392.1
Endogenous	CD45R0	NM_080921.3
Endogenous	CD45RA	NM_002838.4
Endogenous	CD45RB	ENST00000367367.1
Endogenous	CD48	NM_001778.2
Endogenous	CD68	NM_001251.2
Endogenous	CD70	NM_001252.2
Endogenous	CD72	NM_001782.2
Endogenous	CD74	NM_001025159.1
Endogenous	CD79A	NM_021601.3
Endogenous	CD79B	NM_021602.2
Endogenous	CD80	NM_005191.3
Endogenous	CD81	NM_004356.3
Endogenous	CD86	NM_006889.3
Endogenous	CD8A	NM_001768.5
Endogenous	CD8B	NM_172099.2
Endogenous	CDH1	NM_004360.2
Endogenous	CDH2	NM_001792.3
Endogenous	CDH3	NM_001793.4
Endogenous	CEACAM1	NM_001712.3
Endogenous	CEACAM16	NM_001039213.2
Endogenous	CEACAM19	NM_001127893.1

Endogenous	CEACAM20	NM_001102597.1
Endogenous	CEACAM21	NM_001098506.1
Endogenous	CEACAM3	NM_001815.2
Endogenous	CEACAM4	NM_001817.2
Endogenous	CEACAM5	NM_004363.2
Endogenous	CEACAM6	NM_002483.4
Endogenous	CEACAM7	NM_006890.2
Endogenous	CEACAM8	NM_001816.3
Endogenous	CELA1	NM_001971.5
Endogenous	CERCAM	NM_016174.4
Endogenous	CERK	NM_182661.1
Endogenous	CHIT1	NM_003465.2
Endogenous	CHL1	NM_006614.2
Endogenous	CHUK	NM_001278.3
Endogenous	CIITA	NM_000246.3
Endogenous	CKLF	NM_001040138.1
Endogenous	CLEC4E	NM_014358.2
Endogenous	CLEC4M	NM_001144907.1
Endogenous	CLEC6A	NM_001007033.1
Endogenous	CLEC7A	NM_197954.2
Endogenous	CLTC	NM_004859.3
Endogenous	CMA1	NM_001836.2
Endogenous	COX5A	NM_004255.3
Endogenous	CR1	XM_005273064.1
Endogenous	CR2	NM_001877.3
Endogenous	CRADD	NM_003805.3
Endogenous	CRP	NM_000567.2
Endogenous	CSF1R	NM_005211.3
Endogenous	CSRP1	NM_004078.2
Endogenous	CTLA4	NM_005214.3
Endogenous	CTNNB1	NM_001098210.1
Endogenous	CTSB	NM_001908.3
Endogenous	CTSE	NM_001910.2
Endogenous	CTSG	NM_001911.2
Endogenous	CTSL	NM_001912.4
Endogenous	CTSS	NM_004079.3
Endogenous	CTSV	NM_001333.3
Endogenous	CX3CL1	NM_002996.3
Endogenous	CX3CR1	NM_001337.3
Endogenous	CXCL1	NM_001511.1
Endogenous	CXCL10	NM_001565.3
Endogenous	CXCL11	NM_005409.3
Endogenous	CXCL12	NM_000609.5
Endogenous	CXCL13	NM_006419.2
Endogenous	CXCL14	NM_004887.4
Endogenous	CXCL16	NM_001100812.1

Endogenous	CXCL17	NM_198477.1
Endogenous	CXCL2	NM_002089.3
Endogenous	CXCL3	NM_002090.2
Endogenous	CXCL5	NM_002994.3
Endogenous	CXCL6	NM_002993.3
Endogenous	CXCL8	NM_000584.2
Endogenous	CXCL9	NM_002416.1
Endogenous	CXCR1	NM_000634.2
Endogenous	CXCR2	NM_001168298.1
Endogenous	CXCR4	NM_001008540.1
Endogenous	CXCR6	NM_006564.1
Endogenous	CYBA	NM_000101.2
Endogenous	CYBB	NM_000397.3
Endogenous	CYCS	NM_018947.4
Endogenous	CYLD	NM_015247.1
Endogenous	CYR61	NM_001554.3
Endogenous	DAXX	NM_001350.3
Endogenous	DDR1	NM_001954.4
Endogenous	DDR2	NM_001014796.1
Endogenous	DDX17	NM_030881.3
Endogenous	DDX58	NM_014314.3
Endogenous	DDX60	NM_017631.5
Endogenous	DES	NM_001927.3
Endogenous	DHX58	NM_024119.2
Endogenous	DKK2	NM_014421.2
Endogenous	DLL4	NM_019074.3
Endogenous	DSCAM	NM_001389.3
Endogenous	DSTYK	NM_015375.1
Endogenous	DUSP5	NM_004419.3
Endogenous	DUSP6	NM_001946.2
Endogenous	ECD	NM_001135752.1
Endogenous	ECM1	NM_004425.3
Endogenous	ECM2	NM_001393.2
Endogenous	EDN1	NM_001168319.1
Endogenous	EDN2	NM_001956.3
Endogenous	EDN3	NM_000114.2
Endogenous	EED	NM_003797.2
Endogenous	EFEMP1	NM_004105.3
Endogenous	EFEMP2	NM_016938.3
Endogenous	EFNA1	NM_182685.1
Endogenous	EFNA2	NM_001405.3
Endogenous	EFNA3	NM_004952.4
Endogenous	EFNA4	NM_005227.2
Endogenous	EFNA5	NM_001962.2
Endogenous	EFNB1	NM_004429.4
Endogenous	EFNB2	NM_004093.2

Endogenous	EFNB3	NM_001406.3
Endogenous	EGR1	NM_001964.2
Endogenous	EMR1	NM_001974.3
Endogenous	EPHA1	NM_005232.3
Endogenous	EPHA10	NM_173641.2
Endogenous	EPHA2	NM_004431.2
Endogenous	EPHA3	NM_005233.5
Endogenous	EPHA4	NM_004438.3
Endogenous	EPHA5	NM_004439.4
Endogenous	EPHA6	NM_173655.2
Endogenous	EPHA7	NM_004440.2
Endogenous	EPHB1	NM_004441.3
Endogenous	EPHB2	NM_017449.2
Endogenous	EPHB3	NM_004443.3
Endogenous	EPHB4	NM_004444.4
Endogenous	EPHB6	NM_004445.3
Endogenous	ERAP1	NM_001040458.1
Endogenous	ERAP2	NM_022350.1
Endogenous	ESAM	NM_138961.2
Endogenous	ETV1	NM_004956.4
Endogenous	ETV4	NM_001079675.1
Endogenous	ETV5	NM_004454.2
Endogenous	F11R	NM_144503.1
Endogenous	F2R	NM_001992.2
Endogenous	F2RL1	NM_005242.3
Endogenous	FADD	NM_003824.2
Endogenous	FAM19A1	NM_213609.2
Endogenous	FAM19A2	NM_178539.3
Endogenous	FAM19A3	NM_182759.2
Endogenous	FAM19A4	NM_182522.3
Endogenous	FAM69B	NM_152421.3
Endogenous	FAP	NM_004460.2
Endogenous	FAS	NM_152874.1
Endogenous	FASLG	NM_000639.1
Endogenous	FCER1G	NM_004106.1
Endogenous	FCGR2B	NM_001002273.1
Endogenous	FCGR3A	NM_000569.6
Endogenous	FCGRT	NM_004107.3
Endogenous	FCN2	NM_015837.2
Endogenous	FGF10	NM_004465.1
Endogenous	FGF2	NM_002006.4
Endogenous	FGFR1	NM_023110.2
Endogenous	FGFR2	NM_000141.4
Endogenous	FGFR3	NM_000142.4
Endogenous	FGFR4	NM_002011.3
Endogenous	FGFRL1	NM_001004356.2

Endogenous	FLT1	NM_002019.4
Endogenous	FLT3	NM_004119.2
Endogenous	FLT4	NM_182925.4
Endogenous	FOSL1	NM_005438.3
Endogenous	FOXO1	NM_002015.3
Endogenous	FOXO3	NM_001455.2
Endogenous	FOXP3	NM_014009.3
Endogenous	FREM1	NM_144966.5
Endogenous	FREM2	NM_207361.4
Endogenous	FURIN	NM_002569.2
Endogenous	G6PD	NM_000402.3
Endogenous	GAPDH	NM_002046.3
Endogenous	GDF15-C	NM_004864.2
Endogenous	GDF15-N	NM_004864.2
Endogenous	GFI1	NM_001127216.1
Endogenous	GNLY	NM_012483.2
Endogenous	GPC1	NM_002081.2
Endogenous	GPX1	NM_000581.2
Endogenous	GRAP2	NM_004810.2
Endogenous	GRN	NM_002087.2
Endogenous	GUSB	NM_000181.3
Endogenous	GZMA	NM_006144.2
Endogenous	GZMB	NM_004131.4
Endogenous	GZMK	NM_002104.2
Endogenous	HAVCR2	NM_032782.3
Endogenous	HCK	NM_001172129.1
Endogenous	HEPACAM	NM_152722.4
Endogenous	HGF	NM_000601.4
Endogenous	HLA-A	NM_002116.6
Endogenous	HLA-B	NM_005514.6
Endogenous	HLA-C	NM_002117.4
Endogenous	HLA-DMA	NM_006120.3
Endogenous	HLA-DMB	NM_002118.3
Endogenous	HLA-DOA	NM_002119.3
Endogenous	HLA-DOB	NM_002120.3
Endogenous	HLA-DPA1	NM_033554.2
Endogenous	HLA-DPB1	NM_002121.4
Endogenous	HLA-DQA1	NM_002122.3
Endogenous	HLA-DQA2	NM_020056.3
Endogenous	HLA-DQB1	NM_002123.2
Endogenous	HLA-DRA	NM_019111.4
Endogenous	HLA-DRB1	NM_002124.1
Endogenous	HLA-E	NM_005516.4
Endogenous	HLA-F	NM_001098479.1
Endogenous	HLA-G	NM_002127.4
Endogenous	HLA-H	NR_001434.2

Endogenous	HPRT1	NM_000194.2
Endogenous	HSP90AA1	NM_005348.3
Endogenous	HSPA1A	NM_005345.5
Endogenous	ICAM1	NM_000201.1
Endogenous	ICAM2	NM_000873.3
Endogenous	ICAM3	NM_002162.3
Endogenous	ICAM4	NM_001544.3
Endogenous	ICAM5	NM_003259.3
Endogenous	ICOS	NM_012092.2
Endogenous	ICOSLG	NM_015259.4
Endogenous	IDH1	NM_005896.3
Endogenous	IDH3A	NM_005530.2
Endogenous	IDO1	NM_002164.3
Endogenous	IER3	NM_003897.2
Endogenous	IFI30	NM_006332.3
Endogenous	IFIH1	NM_022168.2
Endogenous	IFIT1	NM_001548.3
Endogenous	IFIT1B	NM_001010987.1
Endogenous	IFIT2	NM_001547.4
Endogenous	IFIT3	NM_001031683.2
Endogenous	IFITM1	NM_003641.3
Endogenous	IFNA1	NM_024013.1
Endogenous	IFNA10	NM_002171.1
Endogenous	IFNA14	NM_002172.2
Endogenous	IFNA16	NM_002173.2
Endogenous	IFNA2	NM_000605.3
Endogenous	IFNA21	NM_002175.2
Endogenous	IFNA5	NM_002169.1
Endogenous	IFNA6	NM_021002.1
Endogenous	IFNA7	NM_021057.2
Endogenous	IFNAR1	NM_000629.2
Endogenous	IFNAR2	NM_000874.3
Endogenous	IFNB1	NM_002176.2
Endogenous	IFNE	NM_176891.4
Endogenous	IFNG	NM_000619.2
Endogenous	IFNGR1	NM_000416.1
Endogenous	IFNGR2	NM_005534.3
Endogenous	IFNK	NM_020124.2
Endogenous	IFNL1	NM_172140.1
Endogenous	IFNL2	NM_172138.1
Endogenous	IFNLR1	NM_173065.1
Endogenous	IFNW1	NM_002177.1
Endogenous	IGF1R	NM_000875.3
Endogenous	IGF2R	NM_000876.1
Endogenous	IKBKB	NM_001556.1
Endogenous	IKBKE	NM_014002.2

Endogenous	IKBKG	NM_001099857.1
Endogenous	IKZF1	NM_006060.3
Endogenous	IL10	NM_000572.2
Endogenous	IL10RA	NM_001558.2
Endogenous	IL10RB	NM_000628.3
Endogenous	IL11	NM_000641.2
Endogenous	IL11RA	NM_147162.1
Endogenous	IL12A	NM_000882.2
Endogenous	IL12B	NM_002187.2
Endogenous	IL12RB1	NM_005535.1
Endogenous	IL12RB2	NM_001559.2
Endogenous	IL13	NM_002188.2
Endogenous	IL13RA1	NM_001560.2
Endogenous	IL13RA2	NM_000640.2
Endogenous	IL15	NM_172174.1
Endogenous	IL15RA	NM_002189.2
Endogenous	IL16	NM_004513.4
Endogenous	IL17C	NM_013278.3
Endogenous	IL17D	NM_138284.1
Endogenous	IL17F	NM_052872.3
Endogenous	IL17RA	NM_014339.4
Endogenous	IL17RC	NM_153461.2
Endogenous	IL17RD	NM_017563.3
Endogenous	IL17RE	NM_153483.1
Endogenous	IL18	NM_001562.2
Endogenous	IL18BP	NM_001039659.1
Endogenous	IL18R1	NM_003855.2
Endogenous	IL18RAP	NM_003853.2
Endogenous	IL1A	NM_000575.3
Endogenous	IL1B	NM_000576.2
Endogenous	IL1F10	NM_032556.4
Endogenous	IL1R1	NM_000877.2
Endogenous	IL1R2	NM_173343.1
Endogenous	IL1RAP	NM_002182.2
Endogenous	IL1RL1	NM_016232.4
Endogenous	IL1RL2	NM_003854.2
Endogenous	IL1RN	NM_173841.1
Endogenous	IL2	NM_000586.3
Endogenous	IL20	NM_018724.3
Endogenous	IL20RA	NM_014432.2
Endogenous	IL20RB	NM_144717.2
Endogenous	IL21	NM_021803.2
Endogenous	IL21R	NM_181078.1
Endogenous	IL22	NM_020525.4
Endogenous	IL22RA1	NM_021258.2
Endogenous	IL23A	NM_016584.2

Endogenous	IL23R	NM_144701.2
Endogenous	IL24	NM_001185158.1
Endogenous	IL25	NM_022789.2
Endogenous	IL26	NM_018402.1
Endogenous	IL27	NM_145659.3
Endogenous	IL27RA	NM_004843.2
Endogenous	IL2RA	NM_000417.1
Endogenous	IL2RB	NM_000878.2
Endogenous	IL2RG	NM_000206.1
Endogenous	IL3	NM_000588.3
Endogenous	IL31	NM_001014336.1
Endogenous	IL31RA	NM_139017.3
Endogenous	IL32	NM_001012633.1
Endogenous	IL33	NM_033439.2
Endogenous	IL34	NM_152456.1
Endogenous	IL36A	NM_014440.1
Endogenous	IL36B	NM_014438.3
Endogenous	IL36G	NM_019618.2
Endogenous	IL36RN	NM_012275.2
Endogenous	IL37	NM_014439.3
Endogenous	IL3RA	NM_002183.2
Endogenous	IL4R	NM_000418.2
Endogenous	IL5	NM_000879.2
Endogenous	IL5RA	NM_000564.3
Endogenous	IL6	NM_000600.3
Endogenous	IL6R	NM_000565.2
Endogenous	IL6ST	NM_001190981.1
Endogenous	IL7	NM_000880.2
Endogenous	IL7R	NM_002185.2
Endogenous	IL9	NM_000590.1
Endogenous	IL9R	NM_002186.2
Endogenous	INSR	NM_000208.1
Endogenous	IRAK1	NM_001569.3
Endogenous	IRAK3	NM_007199.1
Endogenous	IREB2	NM_004136.1
Endogenous	IRF1	NM_002198.1
Endogenous	IRF2	NM_002199.2
Endogenous	IRF3	NM_001571.5
Endogenous	IRF4	NM_001195286.1
Endogenous	IRF5	NM_002200.3
Endogenous	IRF6	NM_006147.2
Endogenous	IRF7	NM_004031.2
Endogenous	IRF8	NM_002163.2
Endogenous	IRF9	NM_006084.4
Endogenous	ITGAE	NM_002208.4
Endogenous	ITGAL	NM_002209.2

Endogenous	ITGAM	NM_000632.3
Endogenous	ITGAV	NM_002210.2
Endogenous	ITGAX	NM_000887.3
Endogenous	ITGB2	NM_000211.2
Endogenous	ITK	NM_005546.3
Endogenous	JAM2	NM_021219.2
Endogenous	JAM3	NM_032801.3
Endogenous	KAL1	NM_000216.2
Endogenous	KDR	NM_002253.2
Endogenous	KIT	NM_000222.2
Endogenous	KLF4	NM_004235.4
Endogenous	L1CAM	NM_000425.3
Endogenous	LAG3	NM_002286.5
Endogenous	LBP	NM_004139.2
Endogenous	LCK	NM_005356.3
Endogenous	LCP2	NM_005565.3
Endogenous	LDHA	NM_001165414.1
Endogenous	LEP	NM_000230.2
Endogenous	LEPR	NM_001003679.1
Endogenous	LIFR	NM_001127671.1
Endogenous	LILRB3	NM_006864.2
Endogenous	LIMS1	NM_004987.3
Endogenous	LMBR1	NM_022458.3
Endogenous	LPA	NM_005577.2
Endogenous	LRP1	NM_002332.2
Endogenous	LRRK1	NM_024652.2
Endogenous	LRRK2	NM_198578.2
Endogenous	LTA	NM_001159740.1
Endogenous	LTBP1	NM_000627.3
Endogenous	LTBR	NM_002342.1
Endogenous	LUM	NM_002345.3
Endogenous	LY6G	XM_001475753.3
Endogenous	LY75	NM_002349.2
Endogenous	LY96	NM_015364.2
Endogenous	MADCAM1	NM_130761.1
Endogenous	MAFF	NM_001161572.1
Endogenous	MALT1	NM_006785.2
Endogenous	MAP3K14	NM_003954.1
Endogenous	MAP3K8	NM_005204.2
Endogenous	MARCO	NM_006770.3
Endogenous	MCAM	NM_006500.2
Endogenous	MET	NM_001127500.1
Endogenous	MIA	NM_006533.1
Endogenous	MICA	NM_000247.1
Endogenous	MICB	NM_005931.3
Endogenous	MIEF1	NM_019008.4

Endogenous	MIF	NM_002415.1
Endogenous	MITF	NM_000248.3
Endogenous	MLNR	NM_001507.1
Endogenous	MMD2	NM_198403.3
Endogenous	MMP1	NM_002421.2
Endogenous	MMP11	NM_005940.3
Endogenous	MMP12	NM_002426.3
Endogenous	MMP13	NM_002427.3
Endogenous	MMP14	NM_004995.2
Endogenous	MMP15	NM_002428.2
Endogenous	MMP19	NM_002429.4
Endogenous	MMP2	NM_001127891.1
Endogenous	MMP20	NM_004771.3
Endogenous	MMP21	NM_147191.1
Endogenous	MMP28	NM_001032278.1
Endogenous	MMP3	NM_002422.3
Endogenous	MMP7	NM_002423.3
Endogenous	MMP8	NM_002424.2
Endogenous	MMP9	NM_004994.2
Endogenous	MPL	NM_005373.2
Endogenous	MR1	NM_001531.2
Endogenous	MSR1	NM_138715.2
Endogenous	MUC4	NM_018406.4
Endogenous	MUC5AC	XM_003403450.1
Endogenous	MYD88	NM_001172567.1
Endogenous	NAIP	NM_004536.2
Endogenous	NCAM1	NM_181351.3
Endogenous	NCAM2	NM_004540.2
Endogenous	NCAN	NM_004386.2
Endogenous	NCR1	NM_001242357.1
Endogenous	NDUFS3	NM_004551.1
Endogenous	NFAT5	NM_173214.1
Endogenous	NFATC1	NM_172387.1
Endogenous	NFATC2	NM_173091.2
Endogenous	NFATC3	NM_173164.1
Endogenous	NFATC4	NM_004554.4
Endogenous	NFKB1	NM_001165412.1
Endogenous	NFKBIA	NM_020529.1
Endogenous	NFKBIB	NM_002503.3
Endogenous	NFKBIE	NM_004556.2
Endogenous	NFKBIZ	NM_001005474.1
Endogenous	NFYA	NM_002505.3
Endogenous	NFYB	NM_006166.3
Endogenous	NFYC	NM_001142587.1
Endogenous	NKIRAS1	NM_020345.3
Endogenous	NKIRAS2	NM_001144927.1

Endogenous	NKRF	NM_001173488.1
Endogenous	NLRC3	NM_178844.2
Endogenous	NLRC4	NM_021209.3
Endogenous	NLRP1	NM_033004.2
Endogenous	NLRP10	NM_176821.3
Endogenous	NLRP12	NM_033297.2
Endogenous	NLRP13	NM_176810.2
Endogenous	NLRP2	NM_017852.1
Endogenous	NLRP3	NM_004895.4
Endogenous	NLRP4	NM_134444.4
Endogenous	NLRP6	NM_138329.1
Endogenous	NLRP7	NM_139176.3
Endogenous	NLRP8	NM_176811.2
Endogenous	NLRP9	NM_176820.2
Endogenous	NOD1	NM_006092.1
Endogenous	NOD2	NM_022162.1
Endogenous	NOS1	NM_000620.4
Endogenous	NOS2	NM_000625.4
Endogenous	NOTCH1	NM_017617.3
Endogenous	NOTCH2	NM_024408.3
Endogenous	NRCAM	NM_001037132.1
Endogenous	NRP1	NM_001024629.2
Endogenous	NRP2	NM_003872.2
Endogenous	OGDHL	NM_018245.2
Endogenous	OGFR	NM_007346.2
Endogenous	OLR1	NM_002543.3
Endogenous	P4HA1	NM_000917.3
Endogenous	P4HA2	NM_004199.2
Endogenous	P4HB	NM_000918.3
Endogenous	PDCD1	NM_005018.1
Endogenous	PDCD1LG2	NM_025239.3
Endogenous	PDGFRA	NM_006206.4
Endogenous	PDGFRB	NM_002609.3
Endogenous	PDIA2	NM_006849.2
Endogenous	PDIA3	NM_005313.4
Endogenous	PECAM1	NM_000442.3
Endogenous	PGK1	NM_000291.2
Endogenous	PGLYRP1	NM_005091.1
Endogenous	PGLYRP2	NM_052890.3
Endogenous	PGLYRP3	NM_052891.1
Endogenous	PGLYRP4	NM_020393.2
Endogenous	PJA1	NM_001032396.2
Endogenous	PLA2G2A	NM_000300.2
Endogenous	PLAU	NM_002658.3
Endogenous	PLAUR	NM_002659.2
Endogenous	PLOD1	NM_000302.2

Endogenous	PLOD2	NM_182943.2
Endogenous	PLOD3	NM_001084.4
Endogenous	POLR1B	NM_019014.4
Endogenous	POLR2A	NM_000937.4
Endogenous	PPARG	NM_015869.3
Endogenous	PPARGC1A	NM_013261.3
Endogenous	PPBP	NM_002704.2
Endogenous	PPIA	NM_021130.2
Endogenous	PRF1	NM_005041.3
Endogenous	PRKRIR	NM_004705.2
Endogenous	PROCR	NM_006404.3
Endogenous	PSMB10	NM_002801.2
Endogenous	PSMB8	NM_004159.4
Endogenous	PSMB9	NM_002800.4
Endogenous	PSME1	NM_006263.2
Endogenous	PSME2	NM_002818.2
Endogenous	PSME3	NM_005789.2
Endogenous	PTAFR	NM_000952.3
Endogenous	PTEN	NM_000314.4
Endogenous	PTGS2	NM_000963.1
Endogenous	PTK2	NM_005607.3
Endogenous	PTPRC	NM_080923.2
Endogenous	PTX3	NM_002852.3
Endogenous	PYCARD	NM_013258.3
Endogenous	PYDC1	NM_152901.2
Endogenous	RAC2	NM_002872.3
Endogenous	RARA	NM_001033603.1
Endogenous	RARB	NM_000965.3
Endogenous	RARG	NM_000966.3
Endogenous	RARRES3	NM_004585.3
Endogenous	RBP5	NM_031491.2
Endogenous	REG3G	NM_198448.2
Endogenous	RELA	NM_001145138.1
Endogenous	RELB	NM_006509.2
Endogenous	RFX5	NM_000449.3
Endogenous	ROBO1	NM_002941.2
Endogenous	ROBO2	NM_001128929.1
Endogenous	ROBO3	NM_022370.2
Endogenous	RPL19	NM_000981.3
Endogenous	RPLP0	NM_001002.3
Endogenous	RUNX3	NM_001031680.2
Endogenous	RXFP1	NM_021634.2
Endogenous	S100A1	NM_006271.1
Endogenous	S100A10	NM_002966.1
Endogenous	S100A11	NM_005620.1
Endogenous	S100A12	NM_005621.1

Endogenous	S100A13	NM_001024210.1
Endogenous	S100A14	NM_020672.1
Endogenous	S100A2	NM_005978.3
Endogenous	S100A3	NM_002960.1
Endogenous	S100A5	NM_002962.1
Endogenous	S100A6	NM_014624.3
Endogenous	S100A7	NM_002963.2
Endogenous	S100A7A	NM_176823.3
Endogenous	S100A7L2	NM_001045479.1
Endogenous	S100A8	NM_002964.3
Endogenous	S100A9	NM_002965.2
Endogenous	S100B	NM_006272.1
Endogenous	S100P	NM_005980.2
Endogenous	S100Z	NM_130772.3
Endogenous	S1PR1	NM_001400.3
Endogenous	S1PR2	NM_004230.3
Endogenous	SAA1	NM_199161.1
Endogenous	SAA2	NM_030754.3
Endogenous	SDC1	NM_002997.4
Endogenous	SDC2	NM_002998.3
Endogenous	SDC3	NM_014654.3
Endogenous	SDC4	NM_002999.2
Endogenous	SDHA	NM_004168.2
Endogenous	SDK1	NM_001079653.1
Endogenous	SELL	NR_029467.1
Endogenous	SELP	NM_003005.2
Endogenous	SEMA3A	NM_006080.1
Endogenous	SEMA3B	NM_001005914.1
Endogenous	SEMA3C	NM_006379.2
Endogenous	SEMA3D	NM_152754.2
Endogenous	SEMA3E	NM_012431.1
Endogenous	SEMA3F	NM_004186.3
Endogenous	SEMA3G	NM_020163.1
Endogenous	SEMA4A	NM_001193300.1
Endogenous	SEMA4B	NM_020210.3
Endogenous	SEMA4C	NM_017789.4
Endogenous	SEMA4D	NM_001142287.1
Endogenous	SEMA4F	NM_004263.3
Endogenous	SEMA4G	NM_001203244.1
Endogenous	SEMA5A	NM_003966.2
Endogenous	SEMA5B	NM_001031702.2
Endogenous	SEMA6A	NM_020796.3
Endogenous	SEMA6B	NM_032108.3
Endogenous	SEMA6C	NM_030913.4
Endogenous	SEMA6D	NM_153617.1
Endogenous	SEMA7A	NM_001146029.1

Endogenous	SHFM1	NM_006304.1
Endogenous	SIGLEC14	NM_001098612.1
Endogenous	SIX2	NM_016932.4
Endogenous	SKIV2L	NM_006929.4
Endogenous	SLAMF1	NM_003037.2
Endogenous	SLAMF6	NM_001184714.1
Endogenous	SLAMF7	NM_021181.3
Endogenous	SLIT1	NM_003061.2
Endogenous	SLIT2	NM_004787.1
Endogenous	SLPI	NM_003064.2
Endogenous	SMAD3	NM_005902.3
Endogenous	SMAGP	NM_001033873.1
Endogenous	SMURF2	NM_022739.3
Endogenous	SOCS1	NM_003745.1
Endogenous	SOCS3	NM_003955.3
Endogenous	SOD1	NM_000454.4
Endogenous	SORT1	NM_002959.4
Endogenous	SP1	NM_003109.1
Endogenous	SPI1	NM_001080547.1
Endogenous	SPN	NM_003123.3
Endogenous	SPRY2	NM_005842.2
Endogenous	SPRY4	NM_030964.3
Endogenous	SSTR1	NM_001049.2
Endogenous	SSTR2	NM_001050.2
Endogenous	STAT1	NM_007315.3
Endogenous	STAT3	NM_213662.1
Endogenous	SYK	NM_003177.5
Endogenous	TACR1	NM_015727.1
Endogenous	TANK	NM_004180.2
Endogenous	TAP1	NM_000593.5
Endogenous	TAP2	NM_000544.3
Endogenous	TAPBP	NM_003190.4
Endogenous	TAPBPL	NM_018009.4
Endogenous	TBP	NM_001172085.1
Endogenous	TCF4	NM_001083962.1
Endogenous	TCF7L2	NM_030756.3
Endogenous	TEC	NM_003215.1
Endogenous	TEK	NM_000459.2
Endogenous	TGFB1	NM_000660.4
Endogenous	TGFBR1	NM_001130916.1
Endogenous	TGFBR2	NM_003242.5
Endogenous	TGFBR3	NM_003243.3
Endogenous	THBS1	NM_003246.2
Endogenous	THRA	NM_199334.2
Endogenous	THRB	NM_000461.4
Endogenous	TICAM1	NM_014261.1

Endogenous	TICAM2	NM_021649.4
Endogenous	TIE1	NM_005424.2
Endogenous	TIMP1	NM_003254.2
Endogenous	TIMP2	NM_003255.4
Endogenous	TIMP3	NM_000362.4
Endogenous	TIMP4	NM_003256.2
Endogenous	TK2	NM_004614.3
Endogenous	TLR1	NM_003263.3
Endogenous	TLR10	NM_030956.2
Endogenous	TLR2	NM_003264.3
Endogenous	TLR3	NM_003265.2
Endogenous	TLR4	NM_138554.2
Endogenous	TLR5	NM_003268.3
Endogenous	TLR6	NM_006068.2
Endogenous	TLR7	NM_016562.3
Endogenous	TLR8	NM_016610.2
Endogenous	TLR9	NM_017442.2
Endogenous	TNF	NM_000594.2
Endogenous	TNFAIP3	NM_006290.2
Endogenous	TNFRSF10A	NM_003844.2
Endogenous	TNFRSF10B	NM_147187.1
Endogenous	TNFRSF10C	NM_003841.2
Endogenous	TNFRSF10D	NM_003840.3
Endogenous	TNFRSF11A	NM_003839.2
Endogenous	TNFRSF11B	NM_002546.3
Endogenous	TNFRSF12A	NM_016639.1
Endogenous	TNFRSF13B	NM_012452.2
Endogenous	TNFRSF13C	NM_052945.3
Endogenous	TNFRSF14	NM_003820.2
Endogenous	TNFRSF17	NM_001192.2
Endogenous	TNFRSF18	NM_148901.1
Endogenous	TNFRSF19	NM_018647.3
Endogenous	TNFRSF1A	NM_001065.3
Endogenous	TNFRSF1B	NM_001066.2
Endogenous	TNFRSF21	NM_014452.3
Endogenous	TNFRSF25	NM_148970.1
Endogenous	TNFRSF4	NM_003327.2
Endogenous	TNFRSF6B	NM_003823.2
Endogenous	TNFRSF8	NM_001243.3
Endogenous	TNFRSF9	NM_001561.4
Endogenous	TNFSF10	NR_033994.1
Endogenous	TNFSF11	NM_003701.2
Endogenous	TNFSF12	NM_003809.2
Endogenous	TNFSF13	NM_003808.2
Endogenous	TNFSF13B	NM_006573.4
Endogenous	TNFSF14	NM_003807.2

Endogenous	TNFSF15	NM_005118.2
Endogenous	TNFSF18	NM_005092.2
Endogenous	TNFSF4	NM_003326.2
Endogenous	TNFSF8	NM_001244.2
Endogenous	TNFSF9	NM_003811.3
Endogenous	TP53	NM_000546.2
Endogenous	TRAF3	NM_145725.2
Endogenous	TRIM22	NM_006074.4
Endogenous	TRIM27	NM_006510.4
Endogenous	TRIM46	NM_025058.3
Endogenous	TRIM5	NM_033093.1
Endogenous	TRIM69	NM_080745.3
Endogenous	TRPC4AP	NM_015638.2
Endogenous	TUBB	NM_178014.2
Endogenous	UBR1	NM_174916.2
Endogenous	UBXN1	NM_015853.3
Endogenous	ULBP1	NM_025218.2
Endogenous	ULBP2	NM_025217.2
Endogenous	VCAM1	NM_001078.3
Endogenous	VCAN	NM_004385.3
Endogenous	VEGFA	NM_001025366.1
Endogenous	VIM	NM_003380.3
Endogenous	VTN	NM_000638.3
Endogenous	WNT5A	NM_003392.3
Endogenous	XBP1	NM_005080.2
Endogenous	XCL1	NM_002995.1
Endogenous	XCR1	NM_005283.2
Endogenous	ZAP70	NM_001079.3
Endogenous	ZC3HAV1	NM_020119.3
