# Supplementary material for: Sustained Type I interferon signaling as a mechanism of resistance to PD-1 blockade
Source: Cell Res. 2019 Sep 3;29(10):846–61. doi: 10.1038/s41422-019-0224-x (PMC6796942; doi:10.1038/s41422-019-0224-x)
Supplement: Supplementary file 15 — Table S4: Antibodies [file 41422_2019_224_MOESM15_ESM.pdf]

Table S4: Antibodies

Abs	Color	Clone	Isotype	Company	Ref
Arg1	APC	Polyclonal	Sheep IgG	R&D	IC5868A
CD11b	PB	M1/70	Rat IgG2b	Biolegend	101224
CD11c	PC7	N418	Armenian Hamster IgG	Biolegend	117318
CD11c	BV421	N418	Armenian Hamster IgG	Biolegend	117330
CD3	PC7	145-2C11		BD	552774
CD4	PB	GK1.5		Biolegend	100428
CD4	PE	GK1.5		BD	553730
CD45.2	PerCP Cy5.5	104		eBiosciences	45-0454-82
CD80	PE	16-10A1	Armenian Hamster IgG2	BD	553769
CD86	APC	GL1	Rat IgG2a	BD	558703
CD8a	APC Cy7	53-6.7		BD	557654
CD8a	PerCp	53-6.7		BD	553036
CD8a	FITC	53-6.7		BD	553031
F4/80	APC-Cy7	BM8	Rat IgG2a	Biolegend	123118
Foxp3	APC	FJK-16s	Rat IgG2a	eBiosciences	17-5773-82
Gr1	Pe-Cy7	RB6-8C5	Rat IgG2b	eBiosciences	25-5931-82
H-2Kb	FITC	AF6-120.1	Mouse IgG2a	BD	553551
I-A/I-E	APC	M5/114.15.2	Rat IgG2b	Biolegend	107614
I-A/I-E	PE	M5/114.15.2	Rat IGg2b	eBiosciences	12-5321-82
ICOS	FITC	7E.1769	Armenian Hamster IgG	eBiosciences	11-9942-82
IFNAR 1	APC	MAR1-5A3	Rat IgG2b	Biolegend	127314
IFNg	PerCP Cy5.5	XMG1.2	Rat IgG1k	eBiosciences	45-7311-82
IL-17A	APC	eBio17B7	Rat IgG2ak	eBiosciences	17-7177-81
Ki67	PE	B56	Mouse IgG1	BD	556027
Ki67	FITC	B56	Mouse IgG1	BD	556026
Ly6C	APC Cy7	AL-21	Rat IgM	BD	560596
Ly6G	FITC	1A8	Rat LEW	BD	551460
Nos2	PE	CXNFT	Rat IgG2a	eBiosciences	12-5920-82
PD-1	FITC	J43	Armenian Hamster IgG	eBiosciences	11-9985-85
PD-1	APC	J43	Armenian Hamster IgG	eBiosciences	17-9985-82
PD-L1	PE	MIH5	Rat IgG2a	eBiosciences	12-5982-82
PD-L1	BV421	MIH5	Rat IgG2a	BD	564716
PD-L1	Pe-Cy7	MIH5	Rat IgG2a	eBiosciences	25-5982-82
TNFa	PE	MP6-XT22	Rat IgG1k	eBiosciences	12-7321-82
Viability Dye				Invitrogen	L-34960
